# Assessing Tocolytic Potency: Variability and Accuracy of AUC Versus Amplitude-Based Assessment of Pregnant Human Myometrial Contractions Ex Vivo

**DOI:** 10.1007/s43032-025-01864-0

**Published:** 2025-04-24

**Authors:** Md Reduanul Hossain, Marina Paul, Jorge M. Tolosa, Roger Smith, Jonathan W. Paul

**Affiliations:** 1 College of Health, Medicine and Wellbeing, University of Newcastle, Callaghan, NSW 2308 Australia; 2 Mothers and Babies Research Program, Hunter Medical Research Institute, New Lambton Heights, NSW 2305 Australia; 3 John Hunter Hospital, New Lambton Heights, NSW 2305 Australia

## Abstract

The methodological approaches used to evaluate the inhibitory effects (potency determination) of tocolytic agents on human uterine contractions in ex vivo systems vary, and none of the methodologies has been subjected to scrutiny in terms of objectivity, variability, and reliability. Therefore, this study aimed to assess and compare the reliability of using area under the curve (AUC) versus amplitude alone-based assessment of contraction traces to generate concentration-response curves, which were then used to calculate the tocolytic concentrations that inhibited 25% (IC_25_) and 50% (IC_50_) of baseline contractility. The accuracy of the determined inhibitory concentrations (IC) was then scrutinized while taking into consideration the contraction parameters (amplitude, frequency, duration) affected by the different tocolytics. To do this, pregnant human myometrial strips (term, not-in-labor) were treated with cumulative concentrations of the contraction-blocking agents, nifedipine (NIF), indomethacin (IND), 2-aminoethoxydiphenyl borate (2-APB), glycyl-h-1152 (GH), aminophylline (AMP), or rolipram (ROL)). Concentration-response curves were generated using either AUC or amplitude alone as the index of contraction inhibition, from which tocolytic IC_25_ and IC_50_ concentrations were calculated and compared (i.e. IC_25(AUC)_ vs. IC_25(Amplitude)_). The effects of each tocolytic on contraction frequency were also quantified. To assess accuracy, each tocolytic was applied to contracting strips as a single treatment at their respective IC_25(AUC/Amplitude)_ and IC_50(AUC/Amplitude)_, and then the inhibitory effect re-quantified against both contraction AUC and amplitude alone. Significant differences between IC_50(AUC)_ and IC_50(Amplitude)_ were detected for AMP (318.5 vs. 450 µM), ROL (4.3 vs. 55 µM), and IND (59.5 vs. 75 µM), whereas AUC versus amplitude-based IC_50_ concentrations were comparable for NIF (10 vs. 10 nM), GH (18.2 vs. 15 µM), and 2-APB (53 vs. 57 µM). Similarly, the determined IC_25(AUC)_ and IC_25(Amplitude)_ were again significantly different for AMP (175 vs. 277 µM), ROL (515 nM vs. 15 µM), and IND (28 vs. 42 µM), but also for 2-APB (27 vs. 40 µM). The confirmation studies revealed that a single dose of the tocolytics at their determined IC_25(AUC)_ and IC_50(AUC)_ concentrations consistently reduced contraction AUC by approximately 25% and 50%, respectively, whereas single doses of the IC_25(Amplitude)_ and IC_50(Amplitude)_ concentrations revealed inconsistent results. Of the six tocolytics, four reduced contraction amplitude by > 25% when applied as a single dose at IC_25(Amplitude)_, while three reduced contraction amplitude by > 50% when applied at IC_50(Amplitude)_. Our experimental data indicate that when determining tocolytic potency, assessment of ex vivo contraction traces via AUC is more accurate and consistent than assessment via amplitude alone. Our study finds that whilst tocolytics that increase contraction frequency (while decreasing amplitude) can be assessed by either AUC or amplitude-based determination, tocolytics that either significantly reduce or largely do not affect contraction frequency should only be analyzed via AUC.

**Supplementary Information:**

The online version contains supplementary material available at 10.1007/s43032-025-01864-0.

## Introduction

Preterm birth (PTB) and its sequelae remain major obstetric burdens that affect 15 million babies globally each year and cause significant perinatal mortality and morbidity [1, 2]. Despite improvements in survival rates, preterm neonates are at increased risk of long-term diseases and disabilities, including respiratory diseases, gastrointestinal complications, neurodevelopmental disabilities, and more [3]. The treatment of these morbidities exerts considerable economic and societal expense, as well as familial burden [4, 5]. While the etiology of PTB differs, spontaneous preterm labor has been the leading cause of PTB [2]. Therefore, halting spontaneous preterm labor by pharmacologic inhibition of uterine contractions (tocolysis) continues to be the mainstay of PTB treatment. Tocolytic therapy is intended to reduce neonatal complications by delaying delivery for up to 48 h, which allows time for antenatal corticosteroid administration to improve fetal lung maturation and for maternal transfer to a tertiary care facility [6]. However, existing tocolytics are associated with feto-maternal adverse effects and lack sustained efficacy beyond 48 h [7–9]. Therefore, identifying new tocolytics and their development for PTB prevention are necessary to address this obstetric crisis.

Drug development requires that novel therapeutic compounds be thoroughly tested and evaluated for reliable, robust, and relevant biological effects. The process often involves ex vivo systems that mimic the body’s in vivo physiology of interest. As such, researchers from the disciplines of pharmacology and physiology have relied heavily on isolated tissue organ bath systems for over 100 years [10, 11]. These systems provide researchers with a convenient physiological model that is independent of the systemic influences of the in vivo environment. One such assay is the myometrial contraction assay, where strips of myometrium (uterine smooth muscle) are connected to highly sensitive force transducers and suspended in temperature-controlled organ/tissue baths containing physiological saline [12]. The assay enables the ex vivo determination of whether agents have stimulatory or inhibitory effects on myometrial contractility, including discerning their effects on individual contraction parameters, such as amplitude and frequency, and hence, gaining insight into their therapeutic potential in vivo. As such, the myometrial contraction assay remains a robust pharmacological technique for advancing our understanding of myometrial contractility and developing new therapeutic strategies for addressing complications associated with myometrial dysregulation, including PTB [13–15].

In conducting myometrial contractility studies, contraction data are captured via a data acquisition system (e.g., ADInstruments PowerLab) and visualized and analyzed using its associated software (e.g., LabChart Pro). Contraction traces are analyzed for key contraction parameters, including amplitude/force (g), frequency (number of contractions/h), duration (s), and the integration of these values to determine the area under the curve (AUC) (g tension × s). Despite being a robust assay, confusion can arise because researchers focus on different contraction parameters when assessing the effects of contraction-modulating agents [16–22]. Hence, depending on the contraction parameters analyzed, different potencies may be determined for the same drug. This particularly pertains to drugs that affect contraction amplitude while not affecting the frequency, and vice versa, and has led to accounts in the literature where the therapeutic potency of a particular agent may differ between AUC- or amplitude-based determination. Arrowsmith et al. [21, 22]. demonstrated this in relation to indomethacin and magnesium sulfate (MgSO_4_) treatment of human myometrial tissue strips. The authors showed that when determined for AUC, which incorporates amplitude and frequency, the IC_50_ (the concentration that produces half-maximal inhibition of contraction) for indomethacin [21] and MgSO_4_ [22] were calculated to be 35.4 µM and 1.8 mM, respectively, whereas when determined against amplitude alone, IC_50_ concentrations were calculated to be 45.8 µM and 2.18 mM, respectively. Thus, there is a need to understand which contraction parameters are most appropriate for assessment depending on the drug’s mechanism of action.

The present study aimed to identify the most appropriate approach for determining IC_25_ and IC_50_ concentrations depending upon whether a tocolytic affected amplitude alone, frequency alone, or both amplitude and frequency. To do this, the present study conducted concentration-response analyses for six tocolytics: NIF, a calcium (Ca^2+^) channel blocker [23, 24]; IND, a nonspecific inhibitor of prostaglandin-endoperoxide synthase (PTGS) [25, 26]; 2-APB, a nonspecific inhibitor of inositol triphosphate (IP_3_) [27]; GH, an inhibitor of Rho-associated protein kinase (ROCK) isoenzyme [28]; AMP, a nonspecific phosphodiesterase inhibitor (PDE) [29]; and ROL, a phosphodiesterase 4 inhibitor (PDE4) [20, 30]. These agents were chosen as they have previously been shown to exert differential effects on amplitude and/or frequency, with some agents increasing frequency as amplitude declines, while others decrease or have little effect on frequency as amplitude declines [31]. The location at which these agents act within the myometrial contraction signaling pathway is shown in Supplementary Figure S1.

After determining IC_25_ and IC_50_ concentrations based upon the assessment of amplitude alone, frequency alone, or AUC, follow-up studies were then conducted to assess the accuracy of the determined IC_25_ and IC_50_ concentrations, thus providing insight into which analysis approach should be employed depending upon how a drug affects individual contraction parameters. Additionally, this study draws upon the known literature to provide insight into why one class of drug may affect both contraction amplitude and frequency, whereas other classes may affect amplitude or frequency alone.

## Materials and Methods

### Drugs and Reagents

Drugs and reagents were obtained from the following sources: NIF (cat no. N7634), IND (cat no. I7378), and AMP (cat no. A1755, molecular weight (MW)-210.21) were purchased from Sigma-Aldrich Pty Ltd (Sydney, Australia); ROL (cat no. 0905), GH (cat no. 2485, batch-specific MW 458.3) and 2-APB (cat no. 1224) were purchased from Tocris (Bristol, UK). Other miscellaneous reagents were purchased from Sigma-Aldrich Pty Ltd (Sydney, Australia) and Thermo Fisher Scientific Inc (Sydney, Australia).

### Human Myometrial Biopsy Collection

This study was approved by the Hunter and New England Area Human Research Ethics Committees (2019/ETH12330). Myometrial tissue specimens were obtained with written informed consent from women undergoing elective cesarean delivery at the John Hunter Hospital, NSW, Australia. The tissue specimens were obtained from the upper lip of the incision in the lower uterine segment. All women were between 37 and 40 completed weeks of fetal gestation (term pregnancy) and not-in-labour (NIL). The clinical indications for elective cesarean section were the previous cesarean section, fetal distress, or breech presentation. Upon collection, the myometrial biopsies were placed in pre-chilled phosphate-buffered saline (PBS) on ice and used within 30 min to commence myometrial contraction assays.

### Myometrial Contraction Assays

Myometrial contraction assays were conducted as previously described [31] using an 8-channel temperature-controlled Tissue-Organ Bath System (Radnoti Glass Technology Inc., Monrovia, CA, USA) equipped with MLT0201 force transducers (ADInstruments, Bella Vista, NSW, Australia). Human myometrial samples were dissected into 8 × 1.5 × 1.5 mm strips and then mounted on force transducers (ADInstruments) using stainless-steel tissue clips and nylon thread (ADInstruments). Each strip was lowered into a separate organ bath containing 15 mL modified Krebs-Henseleit buffer (KREBS) (Sigma-Aldrich, cat no. K3753-10 × 1 L) supplemented with 2.5 mM calcium chloride (CaCl_2_) and 25 mM bicarbonate (NaHCO_3_). Organ baths were maintained at 37°C via a circulating water bath and KREBS was continuously gassed with 95% O_2_/5% CO_2_. The transducer position was adjusted to apply 1 g of tension to each strip. Strips were then equilibrated whereby the KREBS was drained and replaced (tissue strips washed) and tension re-applied up to 1 g every 10 min for a total of 30 min. After the third wash/re-tension, the strips were left to develop spontaneous rhythmic contractions ex vivo. Under the experimental setting, myometrial strips took approximately 2 h to establish spontaneous contractions of consistent amplitude and frequency. Contraction data were captured and visualized in real-time using a PowerLab 8/35 data-acquisition system and LabChart software version 8.0 (ADInstruments). Contraction traces were analyzed for key contraction parameters, including amplitude (g), frequency (contractions/h), and integration of these values to determine the AUC (g tension x s). The present study calculated amplitude, frequency, and AUC for all contractions generated during the evaluation/treatment window (outlined below) (Supplementary Figure S2).

### Concentration-Response Study

Before administering drug treatments, a contraction baseline was established for each tissue strip whereby a minimum of 1 h of contractions of consistent amplitude and frequency was recorded. After establishing the baseline, treatments were then added to the organ baths and the effects on contractility were recorded. For each tissue strip, cumulative doses of drugs were administered at 30 min intervals to allow enough time to record therapeutic effects at each concentration. The effect of each drug was assessed against each strip’s contraction baseline (each strip had an internal control). Six drugs (AMP, ROL, IND, NIF, GH, and 2-APB) were analyzed against myometrium from different term pregnant NIL women (replicate numbers as indicated). To control for any effect of drug vehicles (dimethyl sulfoxide (DMSO), water, or KREBS buffer), the equivalent cumulative volume of the appropriate vehicle was tested against a separate strip (different organ bath) during every contraction assay (Supplementary Figure S3 and S4). DMSO was used up to a maximum concentration of 0.42%, which is well below the concentration at which DMSO negatively impacts pregnant human myometrial contractility ex vivo (Supplementary Figure S3).

### Data Analysis

Analyses of the effects of the drugs on contraction amplitude, frequency, and AUC were conducted using LabChart 8.0 Pro with the Dose-Response module (ADInstruments). For each strip, the last 30 min of contractions immediately before commencing the addition of cumulative treatments was measured as the baseline (100%). The effects of treatments were normalized against the baseline, with data expressed as percent (%) of baseline contractility. Concentration-response curves (CRC) for AUC and amplitude were generated using the non-linear regression model of GraphPad Prism 8.0 (GraphPad Software Inc., San Diego, CA, USA) and fitted through the ([log inhibitor] vs. normalized response-variable slope) equation, Y = 100/(1 + 10^((Log IC_50_-X)*Hillslope) (four parameters)). The drug concentration required to inhibit 25% and 50% of the contraction parameter being assessed (i.e. AUC or amplitude), relative to the contraction baseline, was determined as being the IC_25_ and IC_50_, respectively. The frequency of contractions (number of contractions per 30 min of treatment window) was recorded after each dose and normalized against the baseline (number of contractions during the 30 min window immediately before commencing treatments).

### Assessing the Accuracy of the Determined IC_25_ and IC_50_ Concentrations

Following the determination of drug IC_25_ and IC_50_ concentrations, based upon analysis of either AUC or amplitude alone, the present study then sought to assess the accuracy of the determined IC_25_ and IC_50_ concentrations. To do this, baseline contractility for tissue strips was recorded for 1 h. Each drug was then applied to individual contracting strips as a single dose at the IC_25_ and IC_50_ concentrations (separate organ baths and tissue strips for each concentration) determined from analysis of either amplitude or AUC for each drug. Contractility was then recorded for a further 1 h. The effect of administering each drug at the IC_25_ and IC_50_ concentrations was determined for both AUC and amplitude. For assessing the effect of AUC-based IC_25_ and IC_50_ concentrations (IC_25(AUC)_ and IC_50(AUC)_) or amplitude-based IC_25_ and IC_50_ concentrations (IC_25(Amplitude)_ and IC_50(Amplitude)_), the corresponding contraction parameter was used as an index for the measurement of contraction performance. That is, AUC was used as an index when the effects of the IC_25(AUC)_ and IC_50(AUC)_ were assessed, while amplitude was used as an index when the effects of IC_25(Amplitude)_ and IC_50(Amplitude)_ treatment were assessed. In a separate analysis, the effect of IC_25(AUC)_ and IC_50(AUC)_ was assessed on contraction amplitude, and the effect of IC_25(Amplitude)_ and IC_50(Amplitude)_ was assessed on contraction AUC.

### Statistical Analysis

Statistical analyses were conducted with GraphPad Prism software (GraphPad Software Inc., San Diego, CA, USA). All data were checked with the Shapiro-Wilk distribution test for normality. Graphical data are presented as mean ± SEM. For comparisons of multiple groups and interactions, analysis of variance (ANOVA) were conducted. *P*-values < 0.05 were considered statistically significant.

## Results

### Concentration-Response Study

**Fig. 1:**
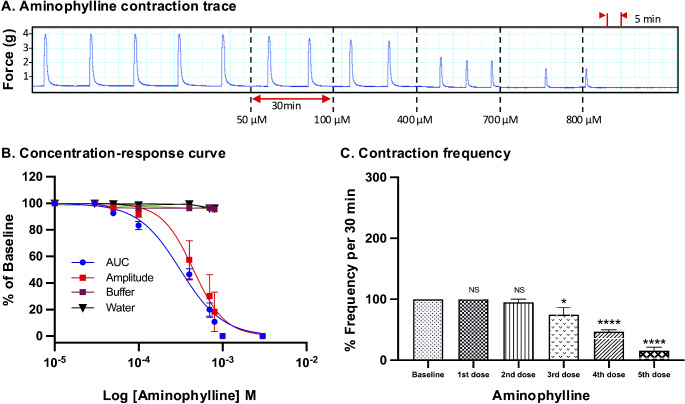
Concentration-response analysis of aminophylline. **(A)** A representative trace for the effect of cumulative treatment of aminophylline (*n* = 8) on spontaneous contractions. Dotted lines indicate the points at which treatments were added to the organ bath. **(B)** The plotted concentration-response curves for the effect of aminophylline on spontaneous pregnant human myometrial contractions ex vivo. Contractility was measured as AUC or amplitude and expressed relative to the contraction baseline (100%). **(C)** The effect of cumulative drug treatments on contraction frequency. The mean percentages of contraction frequency for the cumulative doses are expressed relative to the frequency baselines (100%). Data were checked for normality using the Shapiro-Wilk normality test and then analyzed by 1-way ANOVA with multiple comparisons (Dunnett’s relative to Baseline in all panels). Data are mean ± SEM. ****p ≤ 0.0001; ***p ≤ 0.001; **p ≤ 0.01; *p ≤ 0.05; NS, non-significant

**Fig. 2:**
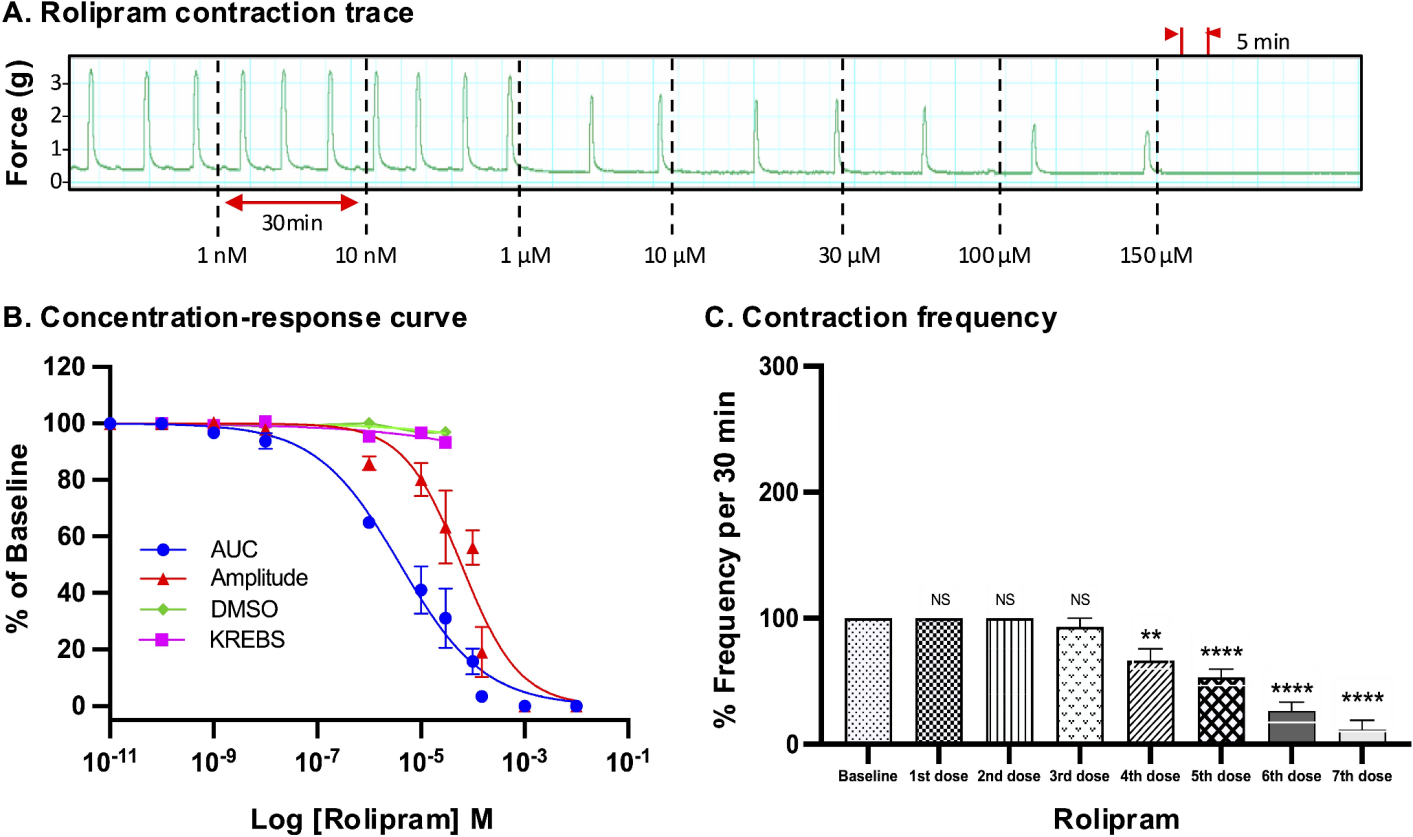
Concentration-response analysis of rolipram. **(A)** A representative trace for the effect of cumulative treatment of rolipram (*n* = 8) on spontaneous contractions. Dotted lines indicate the points at which treatments were added to the organ bath. **(B)** The plotted concentration-response curves for the effect of rolipram on spontaneous pregnant human myometrial contractions ex vivo. Contractility was measured as AUC or amplitude and expressed relative to the contraction baseline (100%). **(C)** The effect of cumulative drug treatments on contraction frequency. The mean percentages of contraction frequency for the cumulative doses are expressed relative to the frequency baselines (100%). Data were checked for normality using the Shapiro-Wilk normality test and then analyzed by 1-way ANOVA with multiple comparisons (Dunnett’s relative to Baseline in all panels). Data are mean ± SEM. ****p ≤ 0.0001; ***p ≤ 0.001; **p ≤ 0.01; *p ≤ 0.05; NS, non-significant

**Fig. 3:**
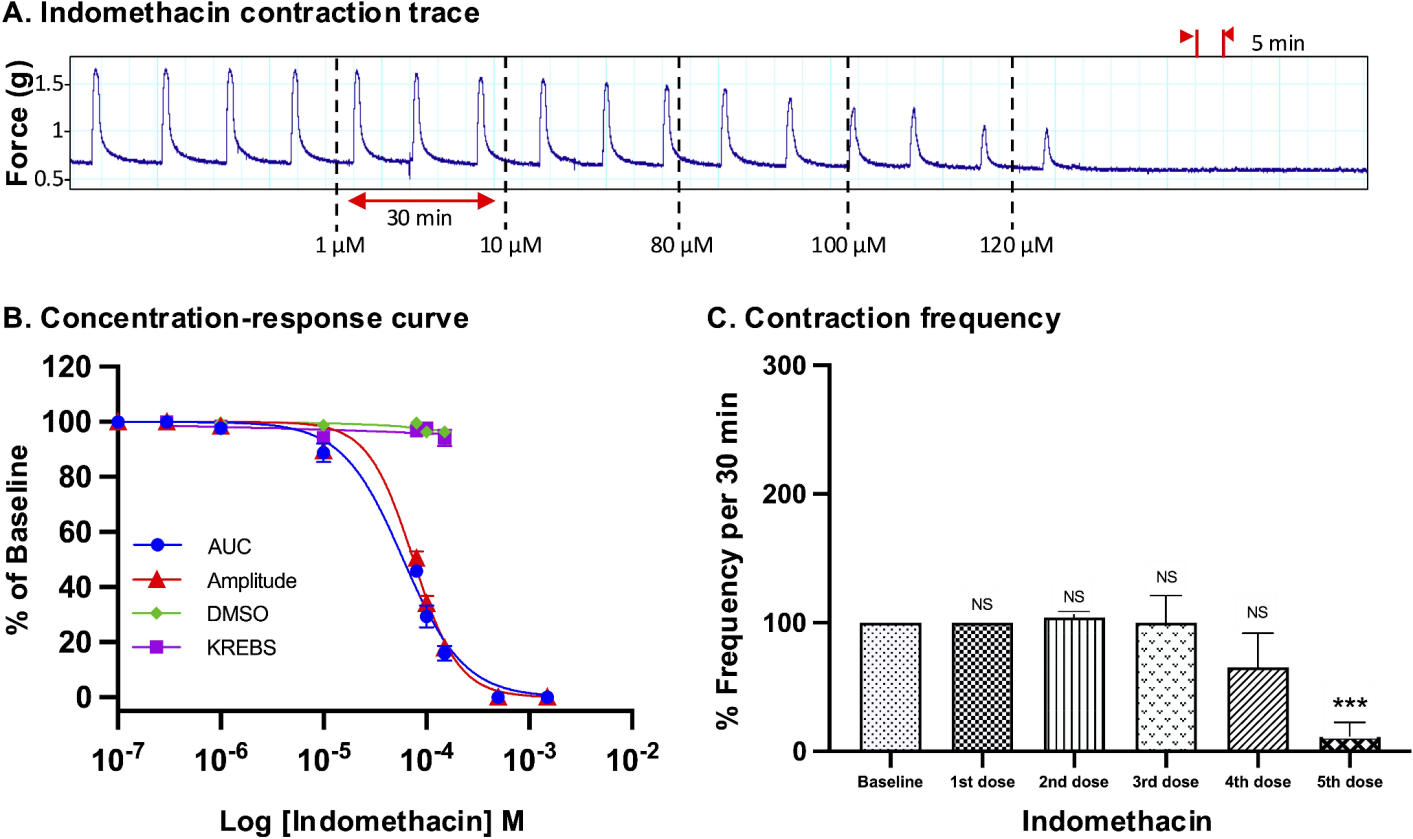
Concentration-response analysis of indomethacin. **(A)** A representative trace for the effect of cumulative treatment of indomethacin (*n* = 10) on spontaneous contractions. Dotted lines indicate the points at which treatments were added to the organ bath. **(B)** The plotted concentration-response curves for the effect of indomethacin on spontaneous pregnant human myometrial contractions ex vivo. Contractility was measured as AUC or amplitude and expressed relative to the contraction baseline (100%). **(C)** The effect of cumulative drug treatments on contraction frequency. The mean percentages of contraction frequency for the cumulative doses are expressed relative to the frequency baselines (100%). Data were checked for normality using the Shapiro-Wilk normality test and then analyzed by 1-way ANOVA with multiple comparisons (Dunnett’s relative to Baseline in all panels). Data are mean ± SEM. ****p ≤ 0.0001; ***p ≤ 0.001; **p ≤ 0.01; *p ≤ 0.05; NS, non-significant

**Fig. 4:**
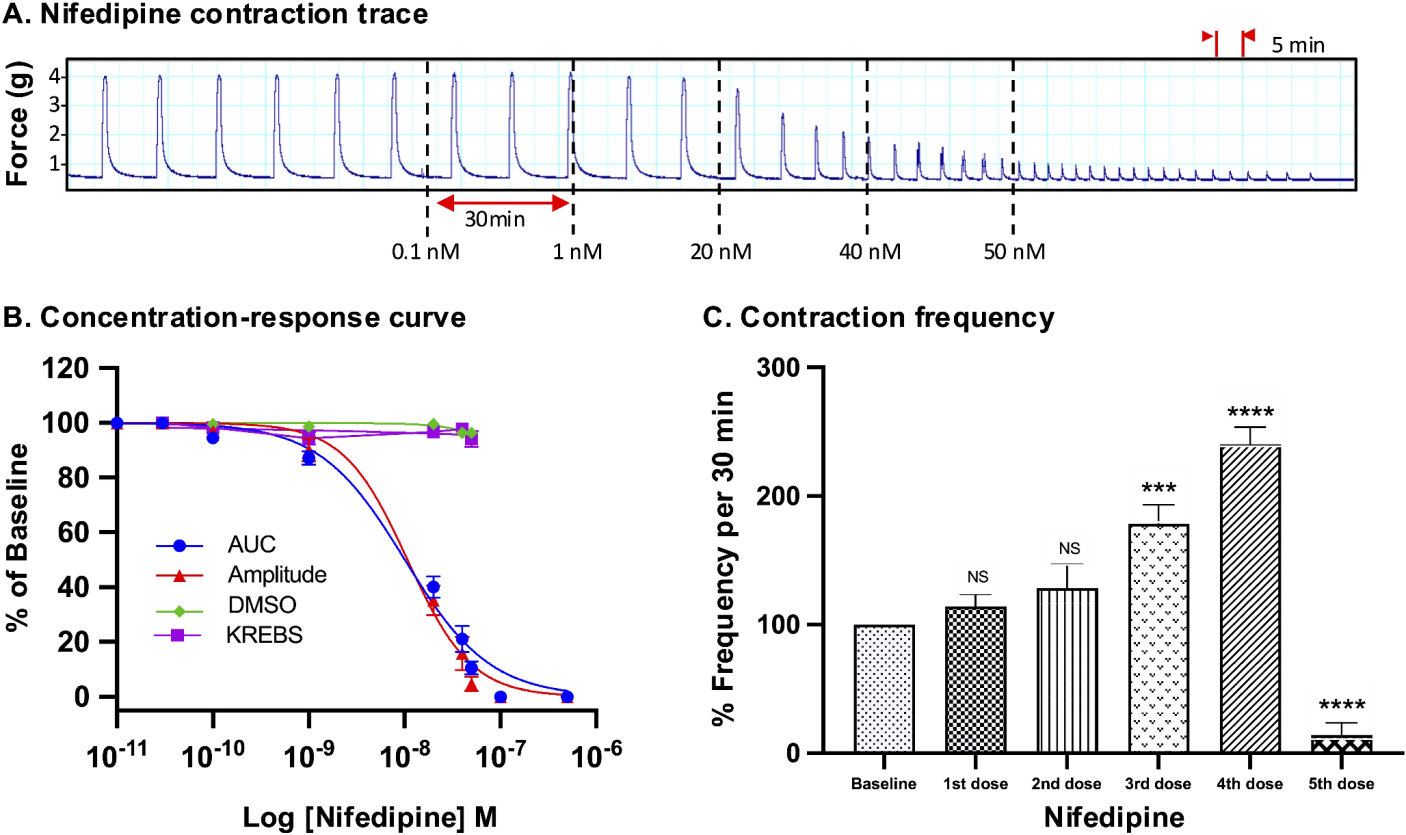
Concentration-response analysis of nifedipine. **(A)** A representative trace for the effect of cumulative treatment of nifedipine (*n* = 10) on spontaneous contractions. Dotted lines indicate the points at which treatments were added to the organ bath. **(B)** The plotted concentration-response curves for the effect of nifedipine on spontaneous pregnant human myometrial contractions ex vivo. Contractility was measured as AUC or amplitude and expressed relative to the contraction baseline (100%). **(C)** The effect of cumulative drug treatments on contraction frequency. The mean percentages of contraction frequency for the cumulative doses are expressed relative to the frequency baselines (100%). Data were checked for normality using the Shapiro-Wilk normality test and then analyzed by 1-way ANOVA with multiple comparisons (Dunnett’s relative to Baseline in all panels). Data are mean ± SEM. ****p ≤ 0.000; ***p ≤ 0.001; **p ≤ 0.01; *p ≤ 0.05; NS, non-significant

**Fig. 5:**
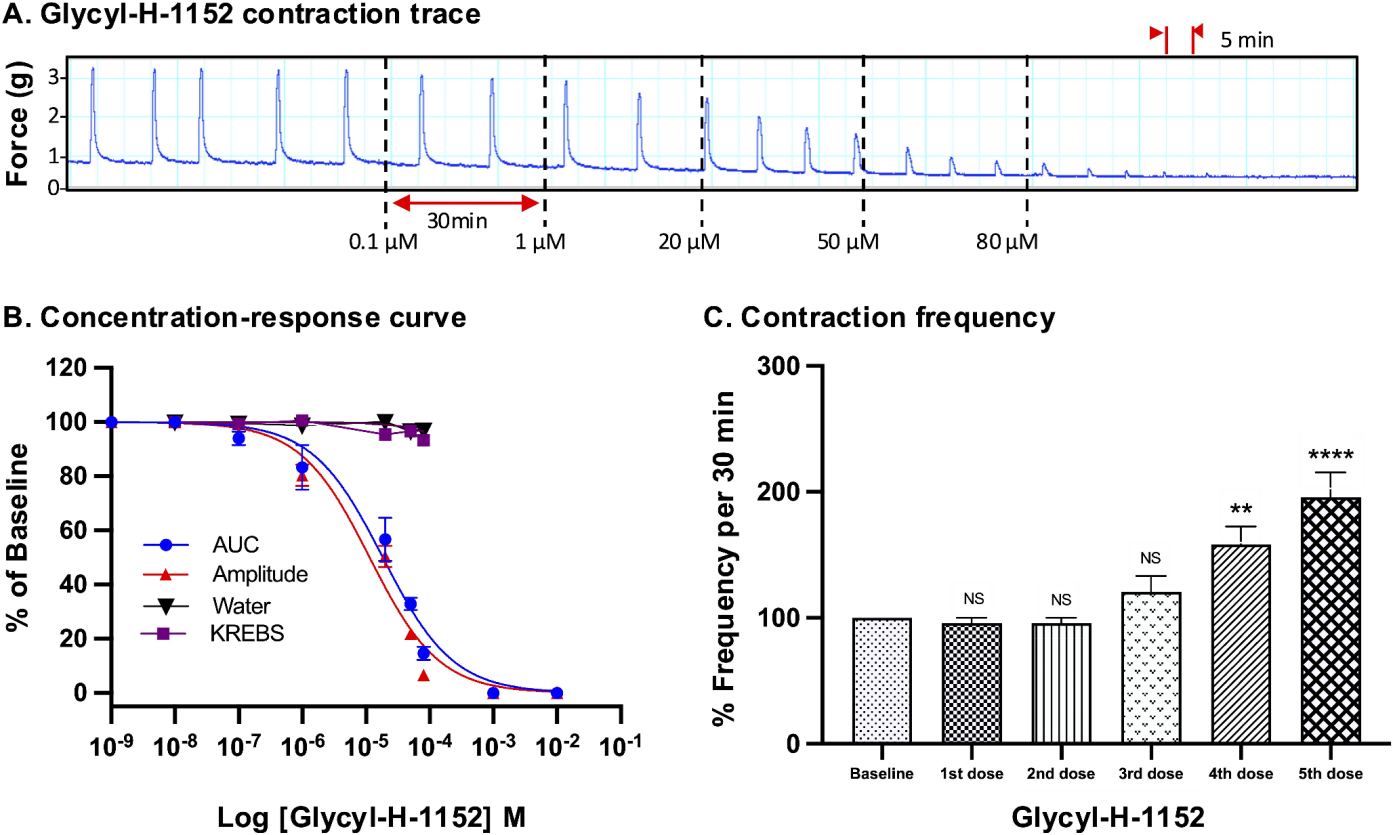
Concentration-response analysis of glycyl-11-52. **(A)** A representative trace for the effect of cumulative treatment of glycyl-H-11-52 (*n* = 8) on spontaneous contractions. Dotted lines indicate the points at which treatments were added to the organ bath. **(B)** The plotted concentration-response curves for the effect of glycyl-11-52 on spontaneous pregnant human myometrial contractions ex vivo. Contractility was measured as AUC or amplitude and expressed relative to the contraction baseline (100%). **(C)** The effect of cumulative drug treatments on contraction frequency. The mean percentages of contraction frequency for the cumulative doses are expressed relative to the frequency baselines (100%). Data were checked for normality using the Shapiro-Wilk normality test and then analyzed by 1-way ANOVA with multiple comparisons (Dunnett’s relative to Baseline in all panels). Data are mean ± SEM. ****p ≤ 0.0001; ***p ≤ 0.001; **p ≤ 0.01; *p ≤ 0.05; NS, non-significant

**Fig. 6:**
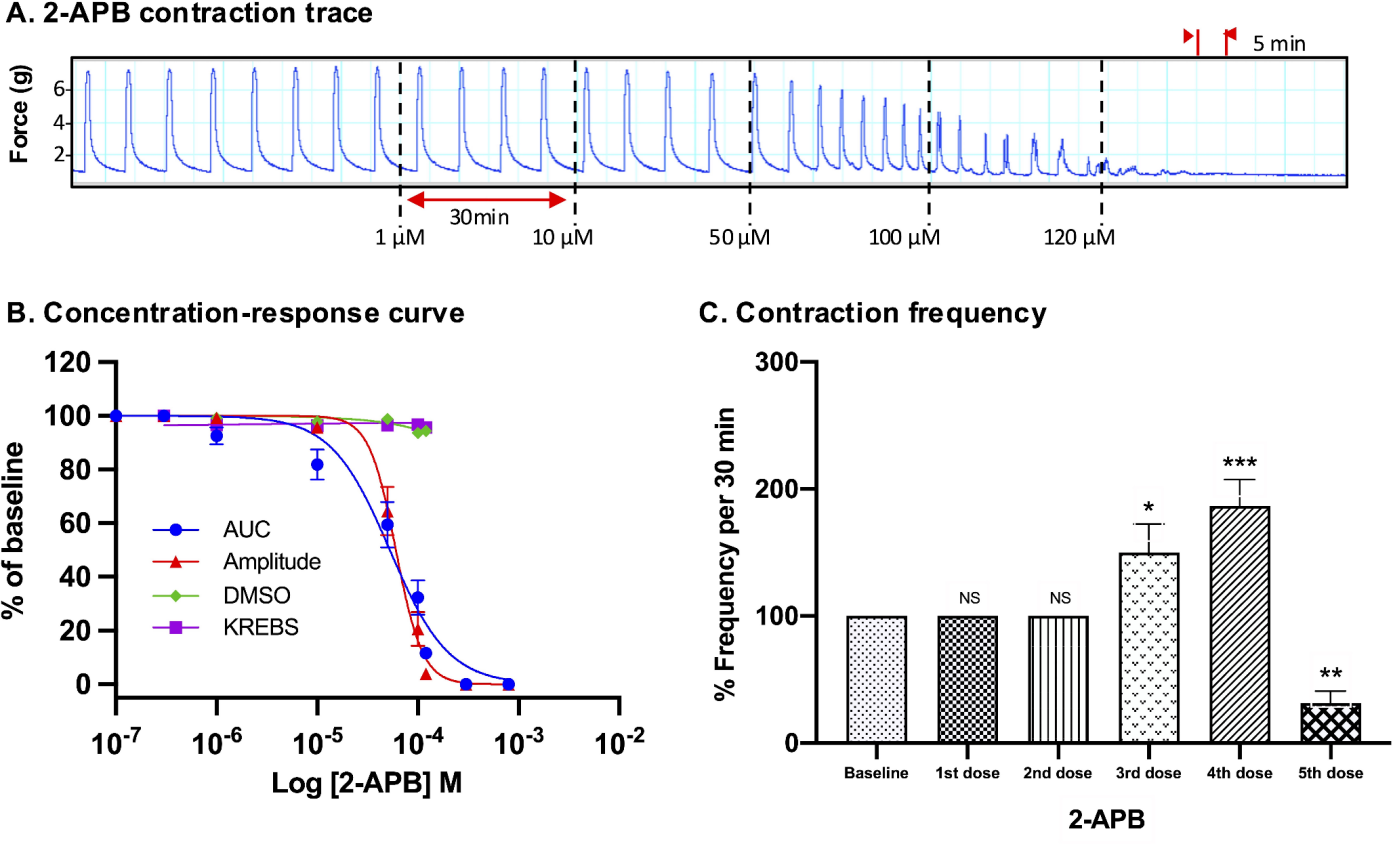
Concentration-response analysis of 2-aminoethoxydiphenyl borate. **(A)** A representative trace for the effect of cumulative treatment of 2-aminoethoxydiphenyl borate (*n* = 8) on spontaneous contractions. Dotted lines indicate the points at which treatments were added to the organ bath. **(B)** The plotted concentration-response curves for the effect of 2-aminoethoxydiphenyl borate on spontaneous pregnant human myometrial contractions ex vivo. Contractility was measured as AUC or amplitude and expressed relative to the contraction baseline (100%). **(C)** The effect of cumulative drug treatments on contraction frequency. The mean percentages of contraction frequency for the cumulative doses are expressed relative to the frequency baselines (100%). Data were checked for normality using the Shapiro-Wilk normality test and then analyzed by 1-way ANOVA with multiple comparisons (Dunnett’s relative to Baseline in all panels). Data are mean ± SEM. ****p ≤ 0.0001; ***p ≤ 0.001; **p ≤ 0.01; *p ≤ 0.05; NS, non-significant

For all drugs, cumulative treatments inhibited spontaneous pregnant human myometrial contractions ex vivo in a concentration-dependent manner. In Figs. 1, 2, 3, 4, 5 and 6, panel A shows representative traces of contractile activity before and after drug treatment. Each drug was assessed against spontaneously contractile tissue strips from *n* = 8–10 women. The CRCs for each drug generated from the corresponding amplitude and AUC measurements are shown in Figs. 1, 2, 3, 4, 5 and 6, panel B, while panel C shows the effect of each drug’s cumulative treatment on contraction frequency. During the treatment courses (2.5–3.5 h), the administration of volume-matched cumulative doses of drug vehicles (DMSO: up to a maximum of 0.42%; Milli-Q water; KREBS) did not affect the contraction amplitude, frequency, or AUC (Supplementary Figures S3 and S4, and Figs. 1, 2, 3, 4, 5 and 6, panel B). The mean IC_25_ and IC_50_ concentrations for each drug, based on their inhibitory effects on contraction amplitude or AUC, are summarized in Table 1. In terms of inhibiting spontaneous pregnant human myometrial contractility ex vivo, when amplitude alone was used as an index, the order of potency of the tocolytics (from highest to lowest) was NIF > GH > ROL > 2-APB > IND > AMP. However, when AUC was used as an index, the order of potency was NIF > ROL > GH > 2-APB > IND > AMP. The order of potency of the tocolytics therefore changed, depending on whether effects on AUC or amplitude alone were used to calculate IC_25_ and IC_50_.

**Table 1:**
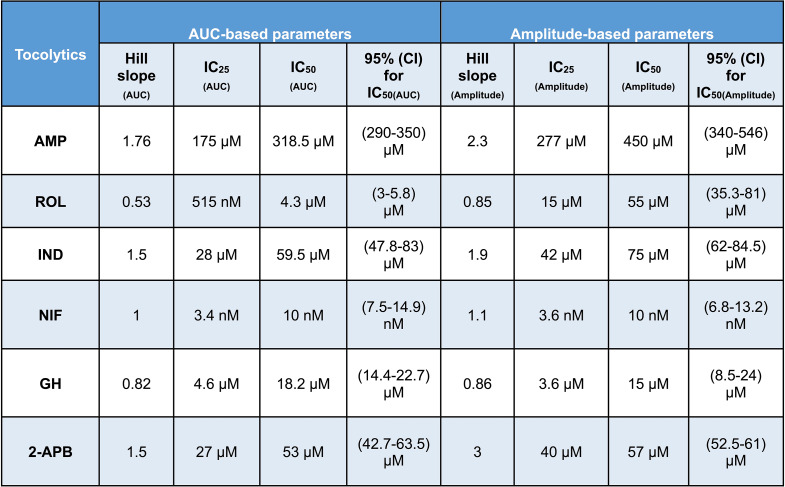
Summary of concentration-response curve parameters for each tocolytic

IC, inhibitory concentration; AUC, area under the curve; AMP, aminophylline; ROL, rolipram; IND, indomethacin; NIF, nifedipine; GH, glycyl-h-1152; 2-APB, 2-aminoethoxydiphenyl borate

### Assessing AUC Versus Amplitude: Effect on IC_25_ and IC_50_

Of the tocolytics analyzed, AMP, ROL, and IND revealed the most marked variations in CRCs when assayed according to amplitude alone versus AUC (Figs. 1, 2 and 3, panels B).

For AMP treatment, the CRCs in Fig. 1, panel B, show that the difference between contraction amplitude and AUC inhibition was evident across the full concentration range. AMP had a more negligible effect on the amplitude and a relatively more prominent effect on contraction AUC (Fig. 1, panel A), which ultimately translated into significant differences in the IC_25_ and IC_50_ concentrations calculated from either AUC or amplitude-based assessment of the contraction traces. For AMP treatment, the IC_25(AUC)_ and IC_25(Amplitude)_ concentrations were determined to be 175 µM and 277 µM, respectively (*p* = 0.04), while the IC_50(AUC)_ and IC_50(Amplitude)_ were determined to be 318.5 µM and 450 µM, respectively (*p* = 0.03) (Tables 1 and 2).

**Table 2:**
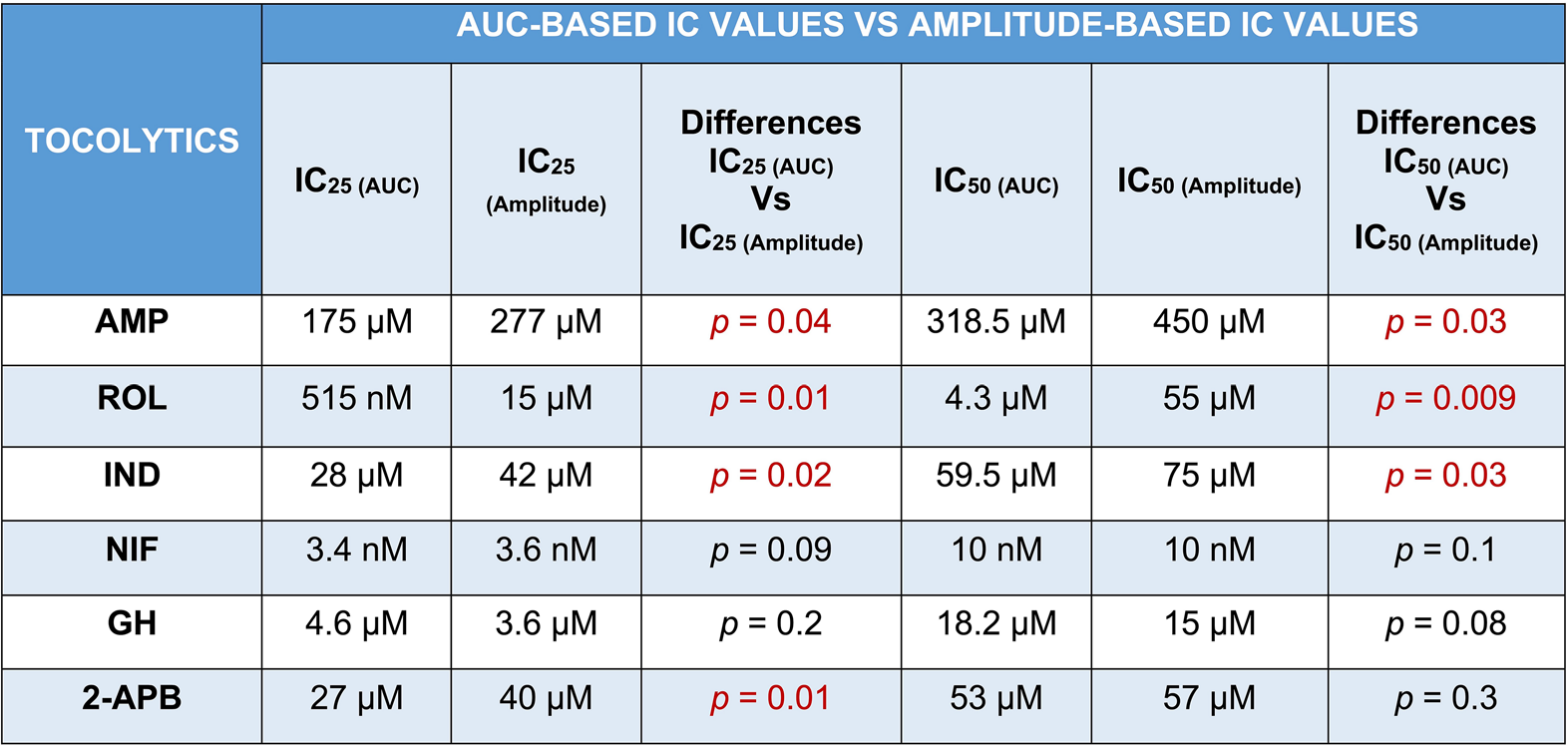
Summary of statistical differences between AUC-based and amplitude-based IC values for each tocolytic

IC, inhibitory concentration; AUC, area under the curve; AMP, aminophylline; ROL, rolipram; IND, indomethacin; NIF, nifedipine; GH, glycyl-h-1152; 2-APB, 2-aminoethoxydiphenyl borate. Data were checked for normality using the Shapiro-Wilk normality test and subsequently analyzed by 1-way ANOVA with multiple comparisons (Dunnett’s relative to Baseline in all panels). Data are presented as mean. Significant p-values (*p*≤0.05) are shown in red text

Consistent findings were observed for ROL treatment on spontaneous contractions, as the IC values were significantly affected according to whether AUC or amplitude was assessed (Fig. 2, panel B). For ROL treatment, the IC_25(AUC)_ and IC_25(Amplitude)_ were determined to be 515 µM and 15 µM, respectively (*p* = 0.01), while the IC_50(AUC)_ and IC_50(Amplitude)_ were determined to be 4.3 µM and 55 µM, respectively (*p* = 0.009) (Tables 1 and 2).

Similarly, the IC values of IND differed according to whether AUC or amplitude was assessed (Fig. 3, panel B). IND inhibited both amplitude and AUC in a dose-dependent manner. The inhibitory effect of IND on contraction amplitude was less pronounced at lower doses, but both amplitude and AUC were affected at higher doses. For IND treatment, the IC_25(AUC)_ and IC_25(Amplitude)_ were determined to be 28 µM and 42 µM, respectively (*p* = 0.025), while the IC_50(AUC)_ and IC_50(Amplitude)_ were determined to be 59.5 µM and 75 µM, respectively (*p* = 0.03) (Tables 1 and 2).

Slight differences were also found between AUC and amplitude-based assessment of the contraction traces for NIF and GH (Figs. 4 and 5, panels A and B). Scrutiny of the CRCs for NIF revealed that contraction AUC and amplitude were almost equally affected across the lower doses (1st and 2nd dose), however, as drug concentration increased, the AUC declined at a shallower slope, due to contraction frequency increasing, which partially offset the loss of amplitude across each 30 min window. However, as the contractions progressively diminished in amplitude, AUC still decreased overall despite the increase in frequency (Fig. 4, panel B). Despite slight differences in the CRC slopes, assessment of NIF treatment contraction traces based on amplitude alone versus AUC did not significantly affect the calculated mean IC_25_ and IC_50_ concentrations. For NIF treatment, the IC_25(AUC)_ and IC_25(Amplitude)_ were calculated to be 3.4 and 3.6 nM, respectively (*p* = 0.09), while the IC_50(AUC)_ and IC_50(Amplitude)_ were 10 nM and 10 nM, respectively (*p* = 0.1) (Tables 1 and 2).

Consistent findings were observed for GH treatment (Fig. 5, panel B), in that the IC_25_ and IC_50_ concentrations determined for GH treatment were not significantly affected by whether AUC or amplitude alone was assessed. For GH treatment, the IC_25(AUC)_ and IC_25(Amplitude)_ were calculated to be 4.6 µM and 3.6 µM, respectively (*p* = 0.2), while the IC_50 (AUC)_ and IC_50(Amplitude)_ were calculated to be 18.2 µM and 15 µM, respectively (*p* = 0.08) (Tables 1 and 2).

The effect of 2-APB on both contraction AUC and amplitude is shown in Fig. 6, panel B. The inhibitory effect on contraction AUC and amplitude was slightly different across the concentration range. At the lower treatment concentrations (1 and 10 µM), 2-APB had little effect on amplitude, however, amplitude declined rapidly at concentrations of 50 µM and above (Fig. 6, panel A), producing the steepest hill slope of all the agents analyzed (Table 1). The shallower hill slope observed for AUC-based assessment of 2-APB’s effects was attributable to 2-APB increasing contraction frequency, in particular at 50 and 100 µM (3rd and 4th doses), which partially offset the rapid loss of contraction amplitude, thus producing a shallower hill slope. Analysis of the CRCs of 2-APB revealed a significant difference between IC_25(AUC)_ and IC_25(Amplitude)_, which were 27 and 40 µM, respectively (*p* = 0.01), however, there was no significant difference between IC_50(AUC)_ and IC_50(Amplitude)_, which were 53 and 57 µM, respectively (*p* = 0.3) (Tables 1 and 2).

The IC_25_ and IC_50_ concentrations determined for each tocolytic by assessment of either AUC or amplitude are summarized in Table 1 and the differences between AUC and amplitude-based IC values are summarized in Table 2.

### Effect of Tocolytics on Contraction Frequency

Panel C in Figs. 1, 2, 3, 4, 5 and 6 shows the effects of the tocolytics on contraction frequency. Treatment with AMP and ROL significantly reduced the frequency of spontaneous pregnant human myometrial contractions ex vivo in a concentration-dependent manner (Figs. 1 and 2, panel C), whereas IND did not significantly affect contraction frequency across the concentration range, with the exception of abolishing contractions at the maximum inhibitory concentration (5th dose) (Fig. 3, panel C). In contrast, NIF, GH, and 2-APB significantly increased contraction frequency as drug concentration increased (Figs. 4, 5 and 6, panel C), highlighting that the tocolytics’ different biochemical mechanisms of action can manifest as markedly different physiological effects on pregnant human uterine contractility.

### AUC Vs. Amplitude: Assessment of Experimentally Determined IC_25_ and IC_50_ Concentrations

**Fig. 7:**
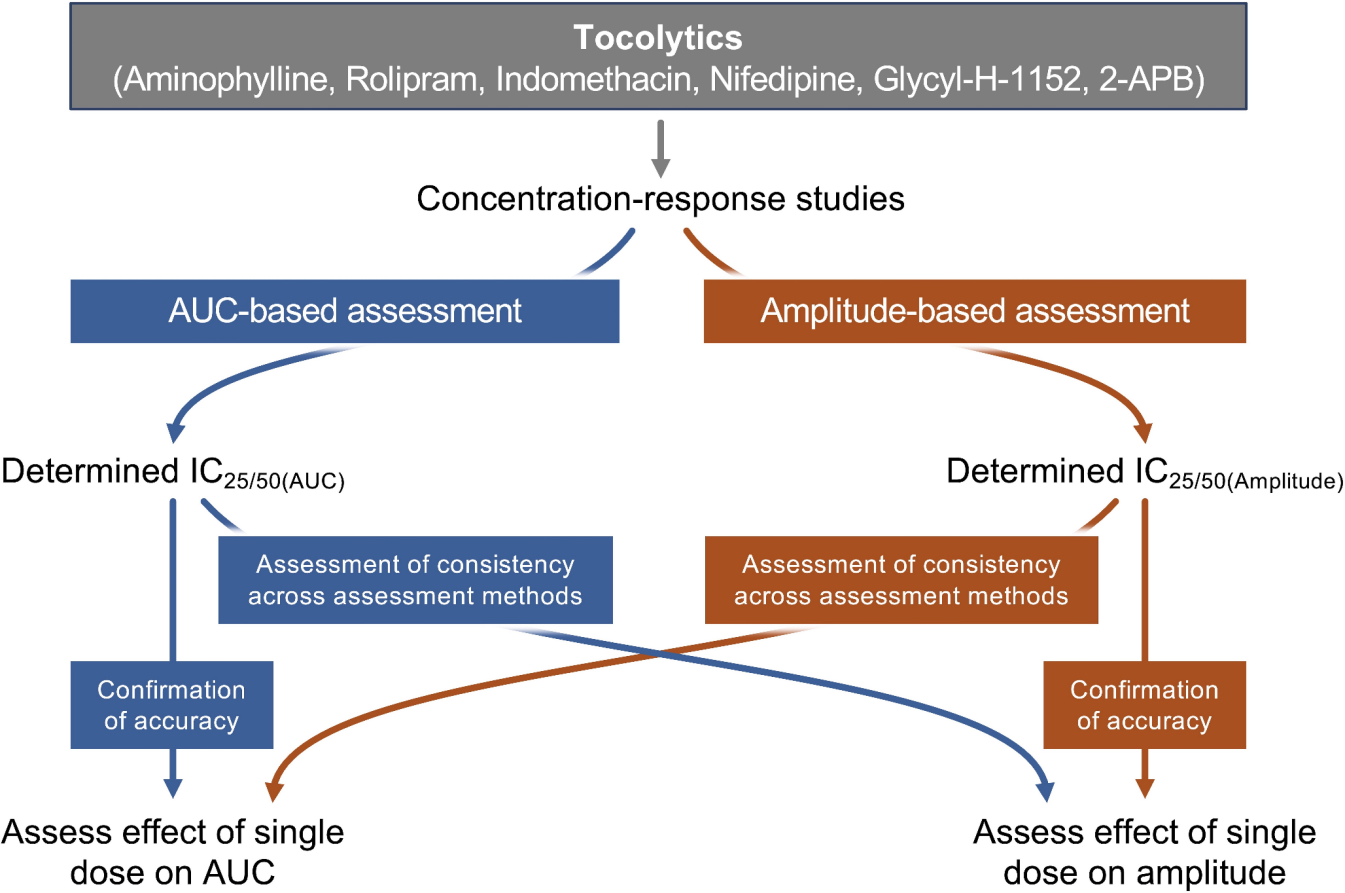
Overview of study layout. The effects of the IC_25_ concentrations were assessed/confirmed for each tocolytic via AUC and amplitude-based assessment. The above experimental layout was repeated for IC_50_ concentrations determined for each of the tocolytics

Having determined the IC_25_ and IC_50_ for each drug based upon assessment of both AUC (IC_25(AUC)_, IC_25(AUC)_) and amplitude (IC_25(Amplitude)_, IC_50(Amplitude)_), follow-up studies were conducted to assess the effects of a single dose of the IC_25/50(AUC)_ concentrations against both AUC and amplitude. This was done to assess/confirm that the IC_25/50(AUC)_ concentrations reduced AUC by 25/50% and to determine whether the AUC-based IC_25/50_ concentrations also reduced amplitude by 25/50%. Similarly, the effects of a single dose of the IC_25/50(Amplitude)_ concentrations were assessed against both AUC and amplitude. This was done to assess/confirm that the IC_25/50(Amplitude)_ concentrations reduced amplitude by 25/50% and to determine whether the amplitude-based IC concentrations also reduced AUC by 25/50%. The study layout for assessing/confirming the effects of the determined IC_25/50_ concentrations is illustrated in Fig. 7.

### Effect of IC_25/50(AUC)_ Concentrations on Contraction AUC and Amplitude

**Fig. 8:**
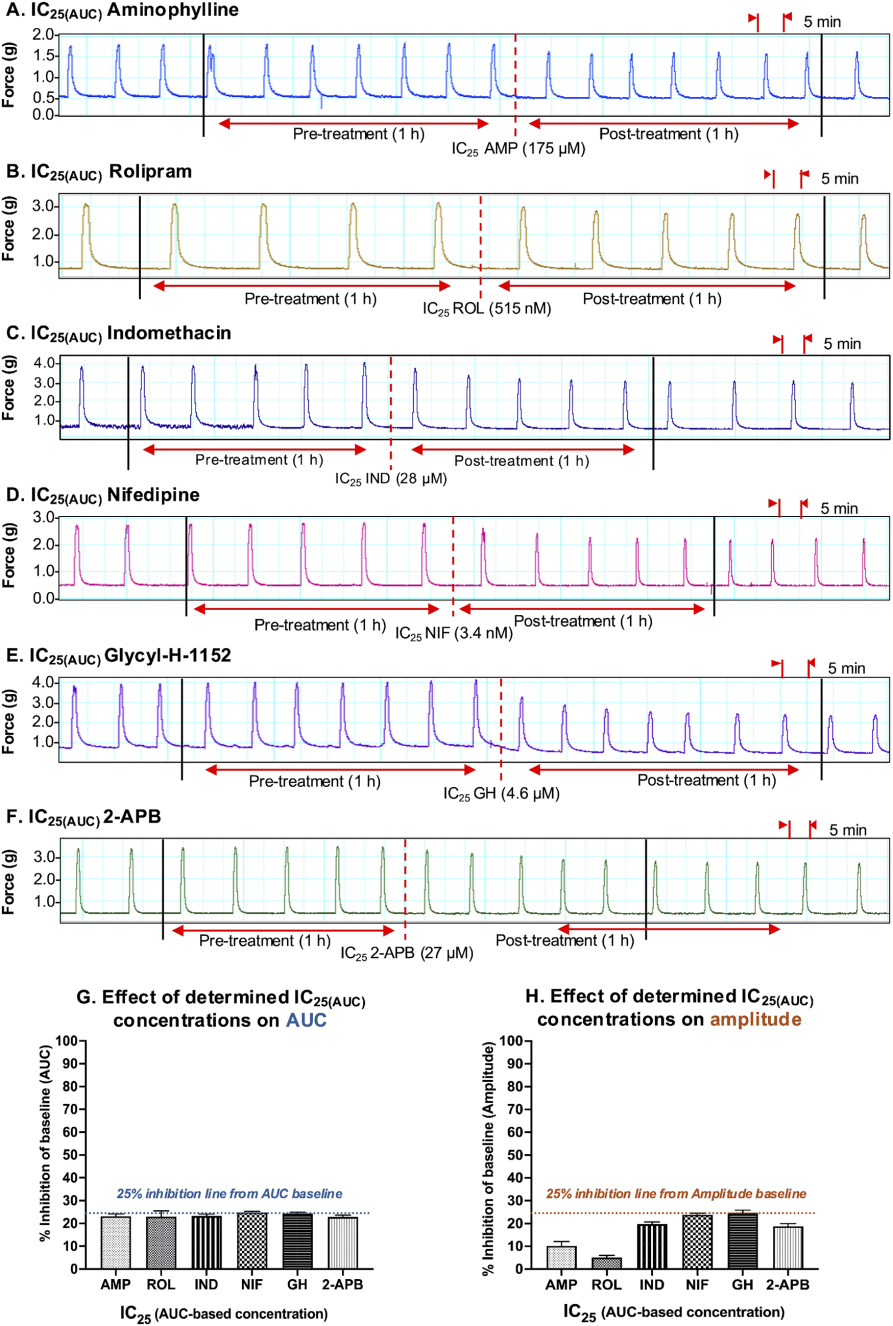
Assessment of the experimentally determined IC_25(AUC)_ concentrations. Representative traces showing the extent of contraction inhibition following treatment of spontaneously contracting pregnant human myometrial strips with the experimentally determined AUC-based IC_25_ concentrations of **(A)** AMP (*n* = 10), **(B)** ROL (*n* = 5), **(C)** IND (*n* = 10), **(D)** NIF (*n* = 10), **(E)** GH (*n* = 10), and **(F)** 2-APB (*n* = 10). Dotted lines indicate the points at which the treatment was added to the organ bath. **(G)** The mean percentage inhibition of AUC (relative to the contraction AUC baselines, 100%) induced by each drug when applied at the IC_25(AUC)_ for 60 min. **(H)** The mean percentage inhibition of amplitude (relative to the contraction amplitude baselines, 100%) induced by each drug when applied at the IC_25(AUC)_ for 60 min. IC, inhibitory concentration; AUC, area under the curve; AMP, aminophylline; ROL, rolipram; IND, indomethacin; NIF, nifedipine; GH, glycyl-h-1152; 2-APB, 2-aminoethoxydiphenyl borate

Looking at the results presented in Fig. 8, when each of the tocolytics was applied as a single dose at the IC_25(AUC)_ concentration, AUC was consistently reduced (1 h pre-treatment vs. 1 h post-treatment) by approximately 25% [AMP = 23.1 ± 1% (*n* = 10); ROL = 23 ± 2.5% (*n* = 5); IND = 23.6% ± 1% (*n* = 10); NIF = 24.8 ± 0.5% (*n* = 10); GH = 24.4 ± 0.5% (*n* = 10); 2-APB = 23 ± 0.8% (*n* = 10)] (Fig. 8, panel G), thus confirming accuracy of the IC_25(AUC)_ concentrations determined during prior concentration-response studies. However, when the effects of the IC_25(AUC)_ were assessed against amplitude, a 25% reduction in amplitude was observed for only two of the six tocolytics. That is, whilst contraction amplitude was indeed reduced by approximately 25% by NIF (23.8 ± 0.7% (*n* = 10)) and GH (24.8 ± 1.1% (*n* = 10)), IND and 2-APB reduced amplitude by 19.8% ± 0.8% (*n* = 10) and 18.8 ± 1.2% (*n* = 10), respectively, whilst AMP and ROL reduced amplitude by just 10.2 ± 1.9% (*n* = 10) and 5.05 ± 0.9% (*n* = 5), respectively (Fig. 8, panel H).

**Fig. 9:**
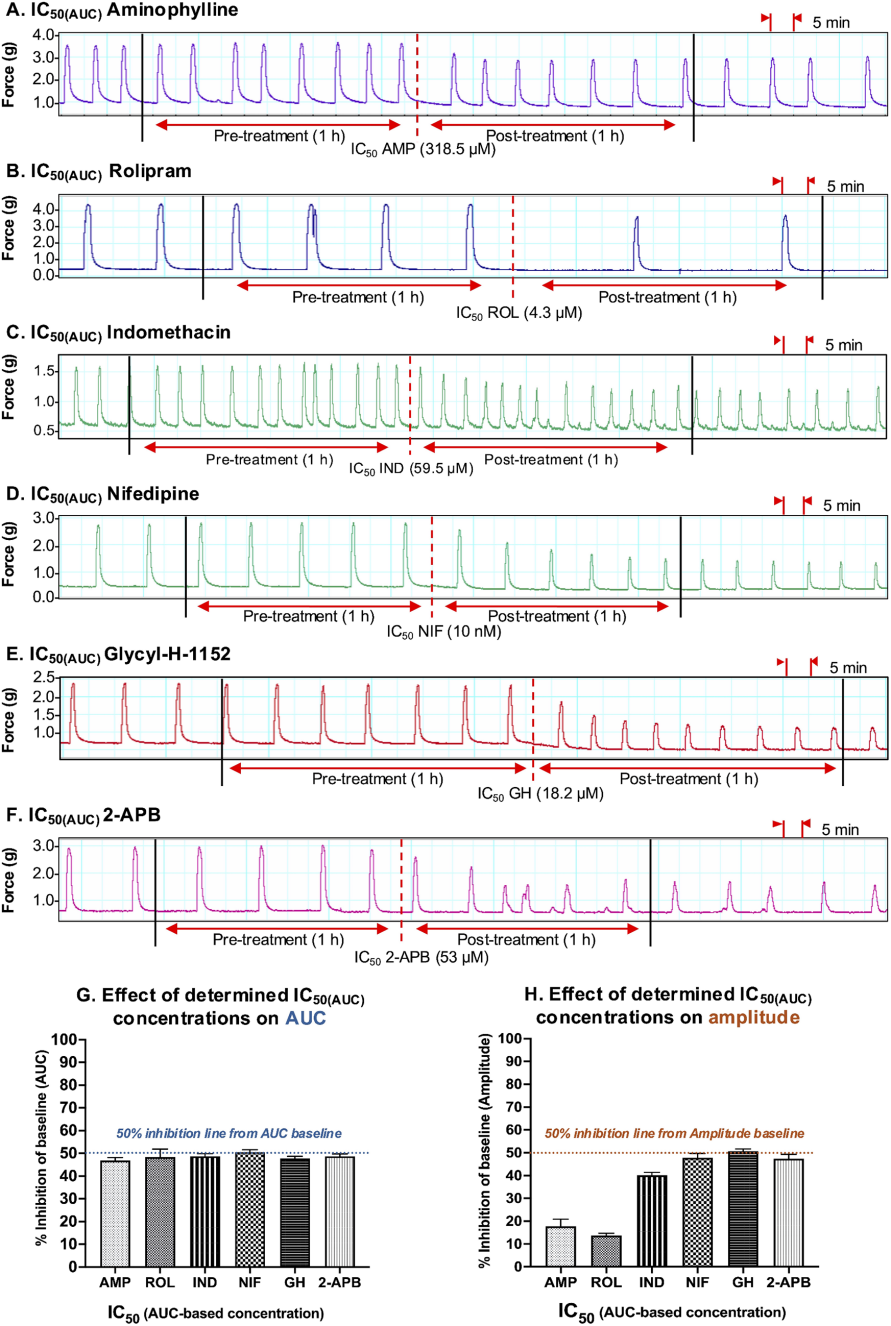
Assessment of the experimentally determined IC_50(AUC)_ concentrations. Representative traces showing the extent of contraction inhibition following treatment of pregnant human myometrial strips with the experimentally determined AUC-based IC_50_ concentrations for **(A)** AMP (*n* = 10), **(B)** ROL (*n* = 5), **(C)** IND (*n* = 10), **(D)** NIF (*n* = 10), **(E)** GH (*n* = 10) and **(F)** 2-APB (*n* = 10). Dotted lines indicate the points at which the treatment was added to the organ bath. **(G)** The mean percentage inhibition of AUC (relative to the contraction AUC baselines, 100%) induced by each drug when applied at the IC_50(AUC)_ for 60 min. **(H)** The mean percentage inhibition of amplitude (relative to the contraction amplitude baselines, 100%) induced by each drug when applied at the IC_50(AUC)_ for 60 min. IC, inhibitory concentration; AUC, area under the curve; AMP, aminophylline; ROL, rolipram; IND, indomethacin; NIF, nifedipine; GH, glycyl-h-1152; 2-APB, 2-aminoethoxydiphenyl borate

The results were largely consistent when each tocolytic was applied as a single treatment but at their respective IC_50(AUC)_ concentrations (Fig. 9). That is, for each tocolytic, the IC_50(AUC)_ consistently reduced AUC by approximately 50% [AMP = 47 ± 1.3% (*n* = 10); ROL = 48 ± 3.5% (*n* = 5); 48.7% ± 1% for IND (*n* = 10); NIF = 50 ± 1% (*n* = 10); GH = 48 ± 0.9% (*n* = 10); 2-APB = 49 ± 1% (*n* = 10)] (Fig. 9, panel G); however, when a single dose of the IC_50(AUC)_ was assessed against amplitude, amplitude was reduced by approximately 50% by NIF (47.8 ± 1.9% (*n* = 10)), GH (50.8 ± 1% (*n* = 10)), and 2-APB (47.4 ± 1.9% (*n* = 10)), but IND reduced amplitude by 40.2% ± 1.4% (*n* = 10), whilst AMP and ROL reduced amplitude by only 17.8 ± 3% (*n* = 10) and 13.8 ± 0.9% (*n* = 5), respectively (Fig. 9, panel H).

### Effect of IC_25/50(Amplitude)_ Concentrations on Contraction Amplitude and AUC

Just as single treatments of the IC_25(AUC)_ and IC_50(AUC)_ concentrations of each tocolytic were assessed against AUC and amplitude, single treatments of the IC_25(Amplitude)_ and IC_50(Amplitude)_ concentrations were also assessed against AUC and amplitude, as indicated in Fig. 7.

**Fig. 10:**
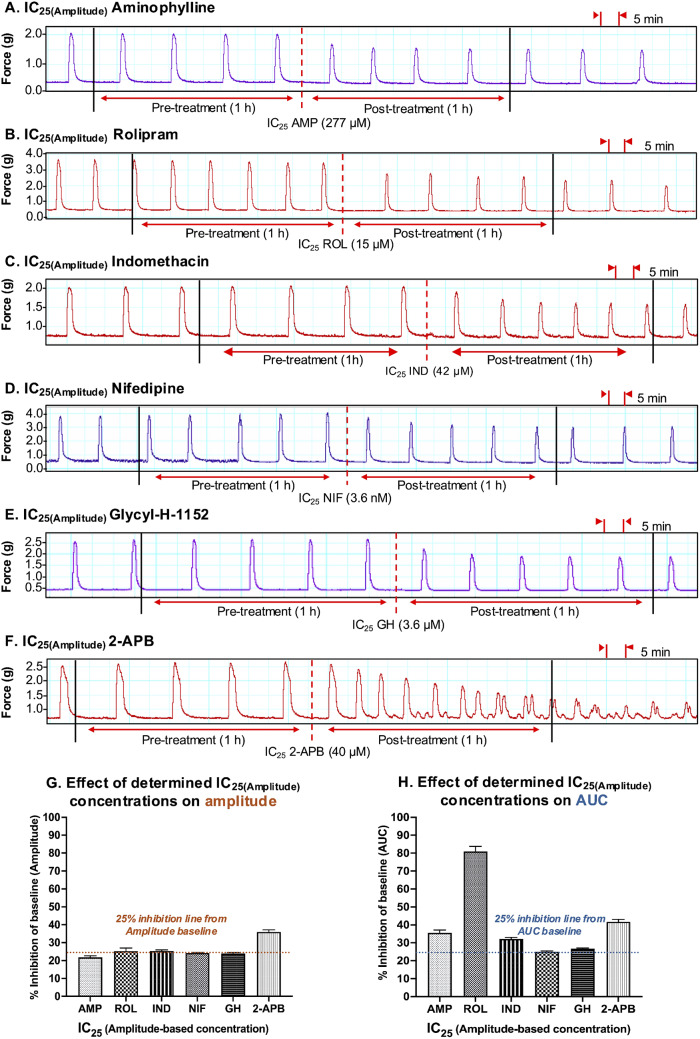
Assessment of the experimentally determined IC_25(Amplitude)_ concentrations. Representative traces showing the extent of contraction inhibition following treatment of pregnant human myometrial strips with the experimentally determined amplitude-based IC_25_ concentrations for **(A)** AMP (*n* = 5), **(B)** ROL (*n* = 5), **(C)** IND (*n* = 5), **(D)** NIF (*n* = 5), **(E)** GH (*n* = 5) and **(F)** 2-APB (*n* = 5). Dotted lines indicate the points at which the treatment was added to the organ bath. **(G)** The mean percentage inhibition of amplitude (relative to the contraction amplitude baselines, 100%) induced by each drug when applied at the IC_25(Amplitude)_ for 60 min. **(H)** The mean percentage inhibition of AUC (relative to the contraction AUC baselines, 100%) induced by each drug when applied at the IC_25(Amplitude)_ for 60 min. IC, inhibitory concentration; AUC, area under the curve; AMP, aminophylline; ROL, rolipram; IND, indomethacin; NIF, nifedipine; GH, glycyl-h-1152; 2-APB, 2-aminoethoxydiphenyl borate

The representative contraction traces in Fig. 10, panels A – F, show the inhibitory effects of a single dose of each tocolytic applied at IC_25(Amplitude)_ concentration (1 h pre-treatment vs. 1 h post-treatment). Amplitude analysis (Fig. 10, panel G) revealed that the IC_25(Amplitude)_ concentration of AMP, ROL, IND, NIF, and GH reduced contraction amplitude by ~ 25% [AMP = 22 ± 1.2% (*n* = 5); ROL = 25.2 ± 1.8% (*n* = 5); IND = 25.3% ± 0.6% (*n* = 5); NIF = 24.2 ± 0.3% (*n* = 5); GH = 24 ± 0.4% (*n* = 5)], as expected, however the IC_25(Amplitude)_ for 2-APB reduced amplitude by 36 ± 1.2% (*n* = 5). Moreover, when the inhibitory effects of the IC_25(Amplitude)_ concentrations were assessed against AUC, the reduction in AUC was not consistently ~ 25%. That is, the observed reduction in AUC was ~ 25% for NIF (25.2 ± 0.5% (*n* = 5)) and GH (26.7 ± 0.4% (*n* = 5)), but the reduction in AUC for the other drugs was greater than 25% [AMP = 35.3 ± 2% (*n* = 5); ROL = 81.1 ± 2.9% (*n* = 5); IND = 32.4% ± 1% (*n* = 5); 2-APB = 41.7 ± 1.4% (*n* = 5)] (Fig. 10, panel H).

**Fig. 11:**
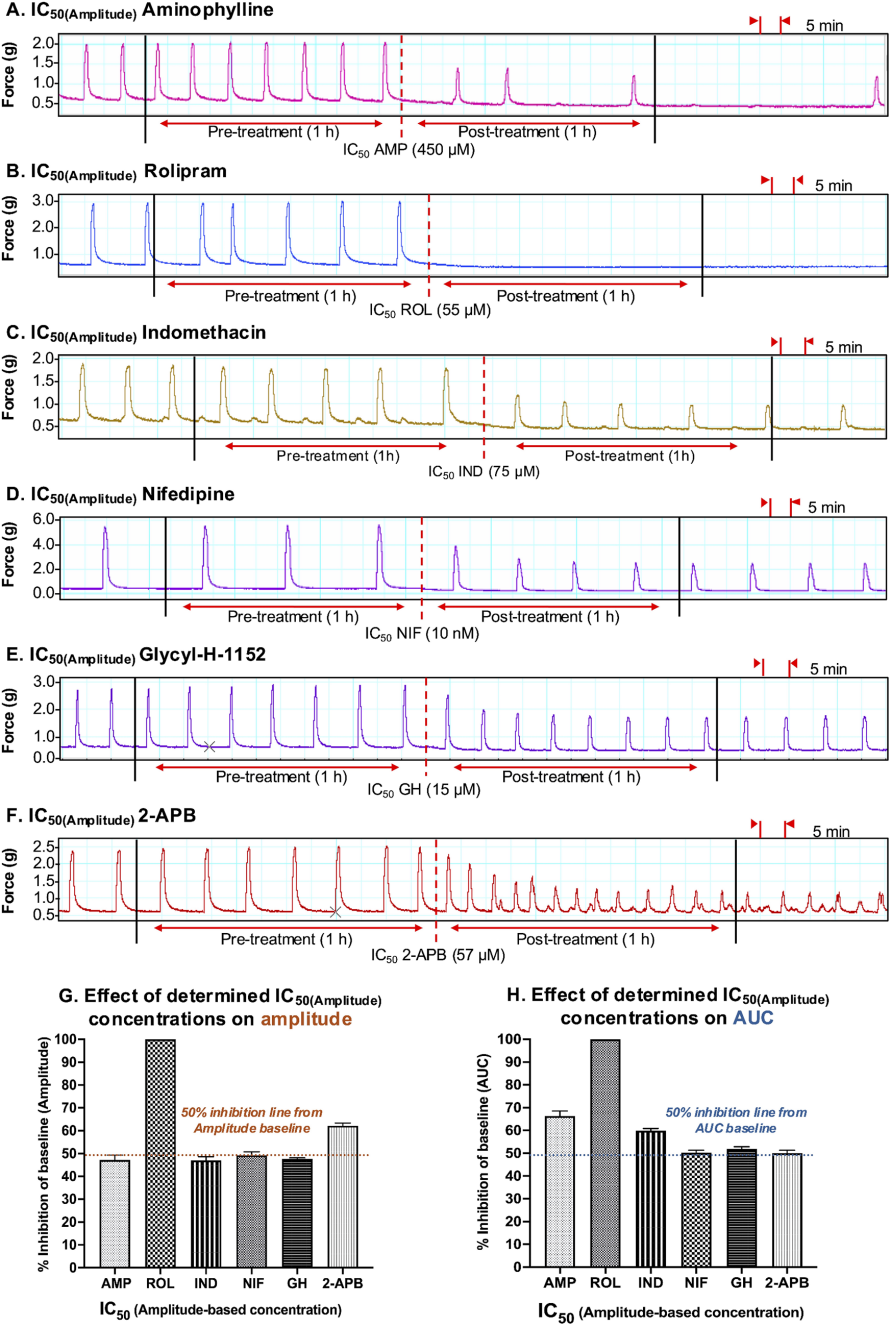
Assessment of the experimentally determined IC_50(Amplitude)_ concentrations. Representative traces showing the extent of contraction inhibition following treatment of pregnant human myometrial strips with the experimentally determined amplitude-based IC_50_ concentrations for **(A)** AMP (*n* = 5), **(B)** ROL (*n* = 5), **(C)** IND (*n* = 5), **(D)** NIF (*n* = 5), **(E)** GH (*n* = 5) and **(F)** 2-APB (*n* = 5). Dotted lines indicate the points at which the treatment was added to the organ bath. **(G)** The mean percentage inhibition of amplitude (relative to the contraction amplitude baselines, 100%) induced by each drug when applied at the IC_50(Amplitude)_ for 60 min. **(H)** The mean percentage inhibition of AUC (relative to the contraction AUC baselines, 100%) induced by each drug when applied at the IC_50(Amplitude)_ for 60 min. IC, inhibitory concentration; AUC, area under the curve; AMP, aminophylline; ROL, rolipram; IND, indomethacin; NIF, nifedipine; GH, glycyl-h-1152; 2-APB, 2-aminoethoxydiphenyl borate

Mostly consistent results were found when each of the tocolytics was applied as a single dose but at their respective IC_50(Amplitude)_ concentrations (Fig. 11). That is, while the IC_50(Amplitude)_ of AMP (47.2 ± 2.5% (*n* = 5)), IND (47.5% ± 2% (*n* = 5)), NIF (49 ± 1.5% (*n* = 5)), and GH (48 ± 1% (*n* = 10)) reduced contraction amplitude by approximately 50%, as expected, ROL and 2-APB reduced amplitude by 100% (*n* = 5) and 62 ± 2% (*n* = 5), respectively (Fig. 11, panel G). Similarly, when the effects of the IC_50(Amplitude)_ were assessed against AUC, a ~ 50% reduction in AUC was observed for NIF (50.2 ± 1% (*n* = 5)), GH (52 ± 1% (*n* = 5)), and 2-APB (50 ± 1.2% (*n* = 5), however, IND and AMP reduced amplitude by ≥60% [IND = 60% ± 1.2% (*n* = 5); AMP = 66.3 ± 2.2% (*n* = 5)], while ROL reduced AUC by 100% (*n* = 5) (Fig. 11, panel H).

The overall observed percent reductions in contraction AUC and amplitude induced by single treatments of the IC_25(AUC)_ and IC_25(Amplitude)_ are summarized in Table 3, while observed percent reductions induced by the IC_50(AUC)_ and IC_50(Amplitude)_ are summarized in Table 4.

**Table 3:**
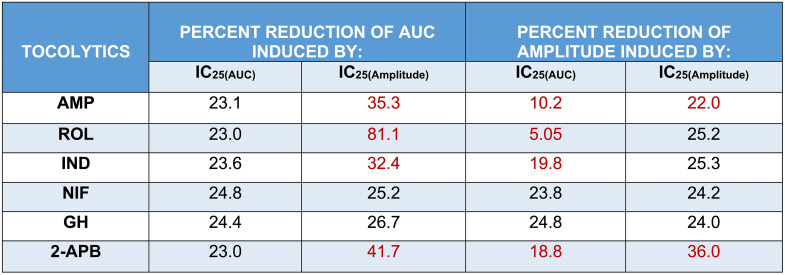
Summary of the percentage reductions of AUC and amplitude induced by the IC_25(AUC)_ and IC_25(Amplitude)_ of each tocolytic

IC, inhibitory concentration; AUC, area under the curve; AMP, aminophylline; ROL, rolipram; IND, indomethacin; NIF, nifedipine; GH, glycyl-h-1152; 2-APB, 2-aminoethoxydiphenyl borate. The red text indicates that the observed percentage reduction was significantly different to the expected reduction of 25% (*p* < 0.05). The data were assessed for normality using the Shapiro-Wilk test and analyzed using a one-way ANOVA with multiple comparisons (Dunnett’s test relative to the expected percentage reduction of 25% across all panels). Data are presented as mean

**Table 4:**
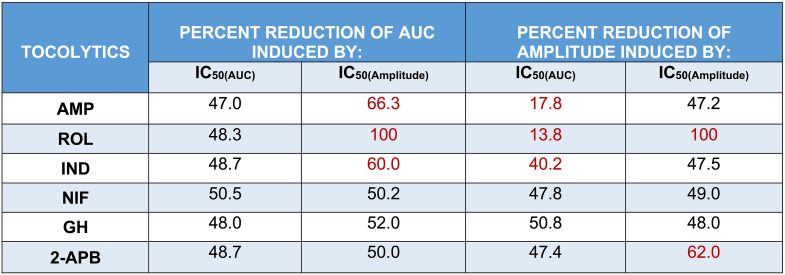
Summary of the percentage reductions of AUC and amplitude induced by the IC_50(AUC)_ and IC_50(Amplitude)_ of each tocolytic

IC, inhibitory concentration; AUC, area under the curve; AMP, aminophylline; ROL, rolipram; IND, indomethacin; NIF, nifedipine; GH, glycyl-h-1152; 2-APB, 2-aminoethoxydiphenyl borate. The red text indicates that the observed percentage reduction was significantly different to the expected reduction of 50% (*p* < 0.05). The data were assessed for normality using the Shapiro-Wilk test and analyzed using a one-way ANOVA with multiple comparisons (Dunnett’s test relative to the expected percentage reduction of 50% across all panels. Data are presented as mean

## Discussion

The present study investigated the reliability of commonly used analysis approaches applied to concentration-response data for assessing tocolytic potency ex vivo [16–22]. In particular, this study found that discrepancies emerged depending on whether tocolytic potency was assessed using AUC or amplitude as the index of contraction performance.

Uterine contractions are composed of three key parameters – amplitude (contraction force), frequency (rate of contraction), and contraction duration. Clinically, all three parameters are crucial to successful labor, as the ability of the uterus to expel the fetus is governed by contraction force and frequency [32]. It is evident from the present data that an individual drug has its characteristic effect on either contraction amplitude, frequency, or both.

### Concentration-Response Analysis

As AUC captures tocolytic effects on contraction amplitude, frequency, and duration, there could be considerable variation in determining drug potency (e.g., IC_25_ and IC_50_) by assessing AUC compared to assessing amplitude alone. In the present study, this was particularly the case for ROL and AMP, which significantly reduced contraction frequency (AMP at 400 µM and ROL at 10 µM) but produced less pronounced reductions in amplitude. The effects of IND on amplitude and frequency were also largely uncoupled but in reverse, compared to ROL and AMP, in that as IND concentration increased, amplitude declined but with almost no accompanying inhibition of contraction frequency (until contractions were abolished). Thus, the amplitude-based CRC shifted right compared to AUC-based CRC in the case of AMP, ROL, and IND, meaning that amplitude inhibition was significantly less than AUC inhibition at a given concentration. Therefore, AMP, ROL, and IND revealed significantly different IC_25_ and IC_50_ concentrations for AUC versus amplitude-based assessment (Tables 1 and 2).

In contrast, NIF and GH showed no significant variation in the potency (IC_25_ and IC_50_ concentrations) between AUC and amplitude-based assessment, as summarized in Tables 1 and 2. As evident in Fig. 4, NIF decreased contraction amplitude in a concentration-dependent manner while simultaneously increasing contraction frequency, with statistical significance reached by 20 nM (3rd concentration). This is consistent with previous ex vivo contraction bioassay studies, where myometrial strips from pregnant and non-pregnant women responded differentially to NIF, with reduced amplitudes and increased frequency at higher doses [16, 17]. A voltage-clamp study on cardiac myocytes found that NIF also inhibited contraction amplitude in this setting, but in contrast, the effects on amplitude were nearly independent of effects on frequency [33]. The reason for the discrepancy is unclear, but likely attributable to the different regulatory mechanisms acting within cardiac versus uterine myocytes. Nonetheless, in the present study, NIF unequivocally increased contraction frequency in a concentration-dependent manner as amplitude decreased. When using AUC as the assessment index, this progressive increase in contraction frequency partially offsets the progressive decrease in amplitude, which caused a divergence between the AUC and amplitude-based CRCs (Fig. 5, panel B). However, as amplitude drops away rapidly, the capacity for increasing frequency to offset the loss of AUC is only minimal. As such, the AUC and amplitude-based CRCs for NIF still largely overlapped across the concentration range, which translated into no significant difference between AUC versus amplitude-based determination of IC concentrations (i.e. IC_25(AUC)_ vs. IC_25(Amplitude)_; IC_50(AUC)_ vs. IC_50(Amplitude)_). As for GH, no prior data was available regarding GH’s effects on contraction amplitude and frequency. In the present study, we report that the effects of GH on myometrial contractility are similar to that of NIF, in that GH-mediated inhibition of contraction amplitude was coupled with a concentration-dependent increase in frequency, with statistical significance reached by 50 µM (4th concentration) (Fig. 5). Moreover, the AUC and amplitude-based CRCs for GH overlapped across the concentration range, which, similar to NIF, led to no significant differences between AUC and amplitude-based IC values.

Treatment with 2-APB, however, revealed an interesting phenomenon. Like NIF and GH, 2-APB inhibited amplitude in a concentration-dependent manner, and this was coupled with a concentration-dependent increase in frequency, with the effect on frequency reaching statistical significance by the 3rd concentration of 50 µM (Fig. 6, panels A and C). This contrasted with Gravina et al. [34], who found that 2-APB decreased contraction frequency at a single concentration of 10 µM. This was consistent with 2nd concentration of 2-APB examined in the present study (10 µM), which had no significant effect on contraction frequency (Fig. 6, panel C). However, unlike NIF and GH, the CRCs of 2-APB revealed mixed outcomes, in that AUC and amplitude-based curves were divergent at the lower and upper concentrations but intersected at approximately the IC_50_ concentration. As such, no significant difference was found between IC_50(AUC)_ and IC_50(Amplitude)_ for 2-APB (53 vs. 57 µM, respectively), however, significant differences were revealed between the IC_25 (AUC)_ and IC_25(Amplitude)_ (27 vs. 40 µM, respectively) (Tables 1 and 2). The divergence is reflected in the differences in hill slope between the AUC and amplitude-based CRCs for 2-APB.

### Hill Slope

**Fig. 12:**
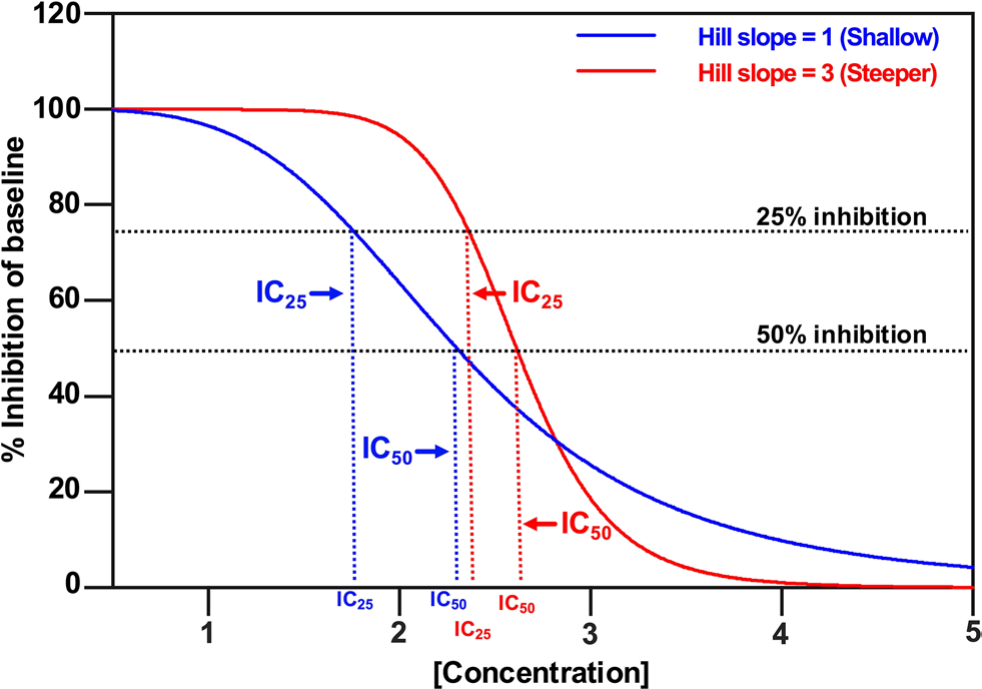
Illustration of two concentration-response curves with different slopes. A typical concentration-response curve is shown in blue. The IC_25_ and IC_50_ are the concentrations that lead to 25% and 50% of the maximal inhibition, respectively. Each curve has a different slope (Hill slope). For the blue curve, the Hill slope = 1, whereas for the red curve, the Hill slope = 3, demonstrating that the higher the Hill slope, the steeper the curve and the higher the IC values (IC_25_ and IC_50_). Hence the IC_25_ and IC_50_ concentrations derived from the two curves are markedly different

Hill slope is a component of a CRC that may be a reliable metric for predicting relevant potency levels [35, 36]. Higher slope values indicate a steeper CRC, while lower slopes indicate a shallower CRC. This affects IC_50_ (and IC_25_) because, at the point of a 50% reduction in either AUC or amplitude (y-axis), steeper curves typically correlate with higher tocolytic concentrations (x-axis) (Fig. 12). For AMP, ROL, and IND treatments, amplitude-based assessment produced CRCs with hill slopes markedly higher than the hill slopes generated from AUC-based assessment (AMP, 2.3 vs. 1.76, respectively; ROL, 0.85 vs. 0.53, respectively; IND, 1.9 vs. 1.5, respectively (Table 1)). These differences in hill slope contributed to the statistically significant differences between the IC_50(AUC)_ and IC_50(Amplitude)_ concentrations determined for AMP, ROL, and IND, as well as between the IC_25(AUC)_ and IC_25(Amplitude)_ concentrations determined for AMP, ROL, and IND (Table 2).

In contrast, NIF and GH revealed nearly equal hill slope values between AUC and amplitude-based determination of the CRCs (NIF, 1 vs. 1.1 respectively; GH, 0.82 vs. 0.86, respectively) (Table 1). The largely consistent hill slopes, derived from either AUC or amplitude-based determination of the CRCs, contributed toward there being no significant differences between the IC_50(AUC)_ and IC_50(Amplitude)_ concentrations for NIF and GH, and likewise between the IC_25(AUC)_ and IC_25(Amplitude)_ concentrations (Table 2).

However, concentration-response analysis for 2-APB revealed a markedly steeper hill slope for amplitude-based CRC determination compared to AUC-based determination (3 vs. 1.5, respectively) (Fig. 6, Panel B and Table 1). Interestingly, there was a significant difference between IC_25(AUC)_ and IC_25(Amplitude)_ concentrations determined for 2-APB (27 vs. 40 µM) (Table 2), which was consistent with differences in hill slope leading to significantly different IC concentrations, as seen for AMP, ROL, and IND; however, for 2-APB the IC_50 (AUC)_ and IC_50 (Amplitude)_ were largely consistent, at 53 vs. 57 µM, respectively (Table 2). This appears to be because although the AUC and amplitude-based curves for 2-APB have markedly different slopes, they just happen to intersect at approximately the IC_50_, leading to largely consistent IC_50_ values from each analysis approach (53 vs. 57 µM) (Fig. 6, panel B and Table 2).

Collectively, these differential effects from individual drugs on contraction parameters and subsequent variances in potency determination raise a question as to which methodological model should be used for the pharmacological analysis of drug effects in the ex vivo system. AUC is a well-established index of contraction performance as it incorporates all key parameters (amplitude, frequency, and duration). Data from the present study also supports the use of AUC for assessing the contraction performance of myometrial tissue strips ex vivo. This is because the confirmation studies showed that for each tocolytic, a single treatment with the IC_25(AUC)_ or IC_50(AUC)_ concentrations (i.e., determined from AUC-based analysis of CRCs) consistently reduced contraction AUC by approximately 25% or 50%, respectively. In contrast, confirmation studies for the IC_25(Amplitude)_ and IC_50(Amplitude)_ concentrations revealed inconsistent results, even when re-assessed against amplitude, despite the IC_25(Amplitude)_ and IC_50(Amplitude)_ having been determined against amplitude. For most drugs, a single treatment of IC_25(Amplitude)_ or IC_50(Amplitude)_ reduced the contraction amplitude by either more than 25% or 50%, respectively, or entirely abolished the contractions. It is noted that the inhibitory effect of IC_AUC_ on amplitude and IC_Amplitude_ on AUC was inconsistent for all drugs, however, the IC_AUC_ was at least consistent when re-assessed against AUC, whereas the IC_Amplitude_ was not consistent when re-assessed against amplitude (Tables 3 and 4).

### Effect of Different Drugs on Contraction Frequency

Finally, another noteworthy observation is that the tocolytics that inhibit myometrial contraction by targeting the Ca^2+^-signaling pathway, namely NIF, IND, 2-APB, and GH, each reduced contraction performance primarily by reducing the contraction strength (amplitude) and either had minimal effect on contraction frequency (lower concentrations) or significantly increased contraction frequency (higher concentrations). In contrast, tocolytics that targeted the cyclic adenosine monophosphate (cAMP)-signaling pathway, namely ROL and AMP, reduced contraction performance primarily by decreasing contraction frequency, with inhibition of contraction amplitude playing a lesser (secondary) role. These differences in physiological effects particularly emerged at the higher concentrations of each drug and may be due to the contribution of ion channels to the slow pacemaker potential and/or resting membrane potential of myocytes that governs the initiation of contractions.

## Conclusion

This study examined the pharmacological effects of six tocolytics on myometrial contraction parameters ex vivo (amplitude, frequency, and AUC). It assessed whether the AUC or amplitude-based determination of IC_25_ and IC_50_ was more appropriate, considering each agent’s effects on amplitude, frequency, and AUC. The study findings quantitatively demonstrated that the different tocolytics differentially affect individual contraction parameters. Consequently, some tocolytics exhibited different concentration-response curves depending on whether AUC or amplitude was used as the assessment index, which translated into significant differences in determined IC concentrations (potency). This tended to be the case for tocolytics that either reduced contraction frequency, such as AMP and ROL, or largely did not affect contraction frequency, such as IND. For other tocolytics, the assessment index did not significantly affect the determined IC_25_ and IC_50_ concentrations. This tended to be the case for tocolytics where the loss of contraction amplitude was coupled with an increase in contraction frequency, such as NIF and GH. Moreover, AUC-based assessment of contraction data revealed less variability, in that IC concentrations determined from AUC were found to be consistently accurate by reconfirmation studies. In contrast, IC concentrations determined by assessment of amplitude alone were not consistently accurate for each tocolytic. In summary, the experimental data herein indicate that (i) AUC is superior to amplitude alone as an index for assessing myometrial contraction performance ex vivo, and (ii) assessing contraction performance via amplitude alone may only be appropriate where a tocolytic increases contraction frequency as amplitude declines.

## Electronic Supplementary Material

Below is the link to the electronic supplementary material.

Supplementary Material 1

Supplementary Material 2

Supplementary Material 3

Supplementary Material 4

## Data Availability

Data are available on request due to privacy/ethical restrictions.

## Abbreviations

2-APB: 2-aminoethoxydiphenyl borate
AMP: Aminophylline
ANOVA: Analysis of variance
ATP: adenosine triphosphate
AUC: area under the curve
BK_Ca_: Large-conductance potassium ion channel
Ca^2+^: Calcium ion
CaCl_2_: Calcium chloride
cAMP: Cyclic adenosine monophosphate
cGMP: Cyclic guanosine monophosphate
DMSO: Dimethyl sulfoxide
CRC: Concentration-response curves
GH: Glycyl-h-1152
HCN: Hyperpolarization-activated cyclic nucleotide-gated channel
HCN4: Potassium/sodium hyperpolarization-activated cyclic nucleotide-gated channel 4
hERG: Human ether-a-go-go related gene channel
IC: Inhibitory concentration
IND: Indomethacin
IP_3_: Inositol triphosphate
K^+^: Potassium ion
K_ATP_: ATP-sensitive potassium ion channel
K_V_: Shaker-like voltage-activated potassium ion channel
MgSO_4_: Magnesium sulfate
MLCK: Myosin light chain kinase
MW: Molecular weight
NaHCO_3_: Bicarbonate
NIF: Nifedipine
NIL: Not-in-labor
PBS: Phosphate-buffered saline
PDE: Phosphodiesterase inhibitor
PDE4: Phosphodiesterase 4 inhibitor
PKA: Protein kinase A
PKG: Protein kinase G
PTB: Preterm birth
PTGS: Prostaglandin-endoperoxide synthase
ROCK: Rho-associated protein kinase
ROL: Rolipram
SK_Ca_: Small conductance calcium-activated potassium channel
TRP: Transient receptor potential

## Declarations

### Persons

We are grateful to the pregnant women who donated myometrial samples, to our research midwife, Ms. Anne Wright, and acknowledge the help of the Obstetrics, Midwifery, and Surgical staff at the John Hunter Hospital, Newcastle.

## References

[1] Chawanpaiboon S, Vogel JP, Moller A-B, Lumbiganon P, Petzold M, Hogan D, Landoulsi S, Jampathong N, Kongwattanakul K, Laopaiboon M, Lewis C, Rattanakanokchai S, Teng DN, Thinkhamrop J, Watananirun K, Zhang J, Zhou W, Gülmezoglu AM. Global, regional, and National estimates of levels of preterm birth in 2014: a systematic review and modelling analysis. Lancet Global Health. 2019;7:e137–46. 10.1016/S2214-109X(18)30451-0. 10.1016/S2214-109X(18)30451-0 PMC6293055 30389451

[2] Goldenberg RL, Culhane JF, Iams JD, Romero R. Epidemiology and causes of preterm birth. Lancet. 2008;371:9606. 10.1016/S0140-6736(08)60074-4. 10.1016/S0140-6736(08)60074-4 PMC7134569 18177778

[3] Saigal S, Doyle LW. An overview of mortality and sequelae of preterm birth from infancy to adulthood. Lancet (London England). 2008;371:9608:261–9. 10.1016/s0140-6736(08)60136-1. 18207020 10.1016/S0140-6736(08)60136-1

[4] Khan KA, Petrou S, Dritsaki M, Johnson SJ, Manktelow B, Draper ES, Smith LK, Seaton SE, Marlow N, Dorling J, Field DJ, Boyle EM. Economic costs associated with moderate and late preterm birth: a prospective population-based study. BJOG: Int J Obstet Gynecol. 2015;122:11. 10.1111/1471-0528.13515. 10.1111/1471-0528.13515 26219352

[5] Petrou S, Yiu HH, Kwon J. Economic consequences of preterm birth: a systematic review of the recent literature (2009–2017). Arch Dis Child. 2019;104:5456. 10.1136/archdischild-2018-315778. 10.1136/archdischild-2018-315778 30413489

[6] ACOG Practice Bulletin: No. 43. May 2003. Management of preterm labor. International Journal of Gynecology & Obstetrics. 2003;82:1:127–135. 10.1016/S0020-7292(03)00247-9 10.1016/s0020-7292(03)00247-9 12834934

[7] Lamont CD, Jørgensen JS, Lamont RF. The safety of tocolytics used for the inhibition of preterm labour. Exp Opin Drug Saf. 2016;15:9:1163–73. 10.1080/14740338.2016.1187128. 10.1080/14740338.2016.1187128 27159501

[8] Berkman ND, Thorp JM Jr., Lohr KN, Carey TS, Hartmann KE, Gavin NI, Hasselblad V, Idicula AE. Tocolytic treatment for the management of preterm labor: A review of the evidence. Am J Obstet Gynecol. 2003;188:6:1648–59. 10.1067/mob.2003.356. 12825006 10.1067/mob.2003.356

[9] Khan K, Zamora J, Lamont RF, Van Geijn Hp H, Svare J, Santos-Jorge C, Jacquemyn Y, Husslein P, Helmer HH, Dudenhausen J, Di Renzo GC, Roura LC, Beattie B. Safety concerns for the use of calcium channel blockers in pregnancy for the treatment of spontaneous preterm labour and hypertension: a systematic review and meta-regression analysis. J Maternal-Fetal Neonatal Med. 2010;23:9:1030–8. 10.3109/14767050903572182. 10.3109/14767050903572182 20180735

[10] Bennett MR. The concept of transmitter receptors: 100 years on. Neuropharmacology. 2000;39:4. 10.1016/S0028-3908(99)00137-9. 10.1016/s0028-3908(99)00137-9 10728874

[11] Loewi O. Über Humorale Übertragbarkeit der herznervenwirkung. Pflüger’s Archiv Für Die Gesamte Physiologie Des Menschen Und Der Tiere. 1921;189(1):239–42.

[12] Arrowsmith S. Human myometrial contractility assays. In: Werry EL, Reekie TA, Kassiou M, editors. Oxytocin: methods and protocols. New York, NY: Springer US. 2022. pp. 29–42. 10.1007/978-1-0716-1759-5_2 10.1007/978-1-0716-1759-5_2 34550566

[13] Paul J, Maiti K, Read M, Hure A, Smith J, Chan EC, Smith R. Phasic phosphorylation of Caldesmon and ERK 1/2 during contractions in human myometrium. PLoS ONE. 2011;6:6e21542. 10.1371/journal.pone.0021542. 10.1371/journal.pone.0021542 PMC3128072 21738699

[14] Paul JW, Hua S, Ilicic M, Tolosa JM, Butler T, Robertson S, et al. Drug delivery to the human and mouse uterus using immunoliposomes targeted to the oxytocin receptor. Am J Obstet Gynecol. 2017;216. 10.1016/j.ajog.2016.08.027. 3:283.e1–283.e14. 10.1016/j.ajog.2016.08.027 27567564

[15] Paul JW, Kemsley JO, Butler TA, Tolosa JM, Thompson MB, Smith R, Whittington CM. A comparison of uterine contractile responsiveness to arginine vasopressin in oviparous and viviparous lizards. J Comp Physiol B. 2020;190:1. 10.1007/s00360-019-01254-4. 31858229 10.1007/s00360-019-01254-4

[16] Forman A, Anderson K-E, Person CGA, Ulmsten U. Relaxant effects of Nifedipine on isolated, human myometrium. Acta Pharmacol Et Toxicologica. 1979;45:2:81–6. 10.1111/j.1600-0773.1979.tb02364.x. 10.1111/j.1600-0773.1979.tb02364.x 495117

[17] Bird LM, Anderson NC Jr., Chandler ML, Young RC. The effects of aminophylline and Nifedipine on contractility of isolated pregnant human myometrium. Am J Obstet Gynecol. 1987;157:1:171–7. 10.1016/S0002-9378(87)80373-3. 3605250 10.1016/s0002-9378(87)80373-3

[18] Berg G, Andersson RGG, Rydén G. In vitro study of phosphodiesterase-inhibiting drugs: A complement to beta-sympathomimetic drug therapy in premature labor? Am J Obstet Gynecol. 1983;145:7:802–6. 10.1016/0002-9378(83)90682-8. 6301281 10.1016/0002-9378(83)90682-8

[19] Berg G, Andersson RG, Ryden G. Effects of different phosphodiesterase-inhibiting drugs on human pregnant myometrium: an in vitro study. Arch Int Pharmacodyn Ther. 1987;290:2288–92. 3446047

[20] Bardou M, Cortijo J, Loustalot C, Taylor S, Perales-Marín A, Mercier FJ, Dumas M, Deneux-Tharaux C, Frydman R, Morcillo EJ, Advenier C. Pharmacological and biochemical study on the effects of selective phosphodiesterase inhibitors on human term myometrium. Naunyn Schmiedebergs Arch Pharmacol. 1999;360:4. 10.1007/s002109900092. 10.1007/s002109900092 10551283

[21] Arrowsmith S, Neilson J, Bricker L, Wray S. Differing in vitro potencies of tocolytics and progesterone in myometrium from Singleton and twin pregnancies. Reproductive Sci. 2016;23(1):98–111. 10.1177/1933719115597788. 10.1177/1933719115597788 26239389

[22] Arrowsmith S, Neilson J, Wray S. The combination tocolytic effect of magnesium sulfate and an oxytocin receptor antagonist in myometrium from singleton and twin pregnancies. Am J Obstet Gynecol. 2016;215. 10.1016/j.ajog.2016.08.015. 6:789.e1–789.e9. 10.1016/j.ajog.2016.08.015 27555315

[23] Fleckenstein A. Specific pharmacology of calcium in myocardium, cardiac pacemakers, and vascular smooth muscle. 1977;17:149–66. 10.1146/annurev.pa.17.040177.001053 10.1146/annurev.pa.17.040177.001053 326161

[24] Wray S, Jones K, Kupittayanant S, Li Y, Matthew A, Monir-Bishty E, Noble K, Pierce SJ, Quenby S, Shmygol AV. Calcium signaling and uterine contractility. J Soc Gynecologic Investigation: JSGI. 2003;10:5:252–64. 10.1016/S1071-5576(03)00089-3. 10.1016/s1071-5576(03)00089-3 12853086

[25] Landen CN, Zhang P, Young RC. Mechanisms of indomethacin and nimesulide on inhibition of calcium rises in human uterine myocytes. Obstet Gynecol. 2001;97(4)Supplement 1:S6. 10.1016/S0029-7844(01)01143-7 10.1067/mob.2001.114915 11349169

[26] Vane JR, Williams KI. The contribution of prostaglandin production to contractions of the isolated uterus of the rat. Br J Pharmacol. 1973;48:4:629–39. 10.1111/j.1476-5381.1973.tb08250.x. 4788206 10.1111/j.1476-5381.1973.tb08250.x PMC1776150

[27] Maruyama T, Kanaji T, Nakade S, Kanno T, Mikoshiba K. 2APB, 2-aminoethoxydiphenyl borate, a membrane-penetrable modulator of ins(1,4,5)P3-induced Ca2 + release. J Biochem. 1997;122:3:498–505. 10.1093/oxfordjournals.jbchem.a021780. 9348075 10.1093/oxfordjournals.jbchem.a021780

[28] Hudson CA, Heesom KJ, López Bernal A. Phasic contractions of isolated human myometrium are associated with Rho-kinase (ROCK)-dependent phosphorylation of myosin phosphatase-targeting subunit (MYPT1). Mol Hum Reprod. 2011;18:5. 10.1093/molehr/gar078. 10.1093/molehr/gar078 PMC3339637 22155728

[29] Coutinho EM, Lopes ACV. Inhibition of uterine motility by aminophylline. Am J Obstet Gynecol. 1971;110:5:726–9. 10.1016/0002-9378(71)90261-4. 4327435 10.1016/0002-9378(71)90261-4

[30] Leroy M-J, Lugnier C, Merezak J, Tanguy G, Olivier S, Le Bec A, Ferré F. Isolation and characterization of the rolipram-sensitive Cyclic AMP-specific phosphodiesterase (type IV PDE) in human term myometrium. Cell Signal. 1994;6:4:405–12. 10.1016/0898-6568(94)90087-6. 7946965 10.1016/0898-6568(94)90087-6

[31] Hossain MR, Tolosa JM, Young RC, Smith R, Paul JW. Assessing the potency of the novel tocolytics 2-APB, Glycyl-H-1152, and HC-067047 in pregnant human myometrium. Reprod Sci. 2023;30:1. 10.1007/s43032-022-01000-2. 35715551 10.1007/s43032-022-01000-2 PMC9810572

[32] Aguilar HN, Mitchell BF. Physiological pathways and molecular mechanisms regulating uterine contractility. Hum Reprod Update. 2010;16:6:725–44. 10.1093/humupd/dmq016. 20551073 10.1093/humupd/dmq016

[33] Bayer R, Rodenkirchen R, Kaufmann R, Lee JH, Hennekes R. The effects of Nifedipine on contraction and monophasic action potential of isolated Cat myocardium. Naunyn Schmiedebergs Arch Pharmacol. 1977;301(1):29–37. 10.1007/BF00501261. 600318 10.1007/BF00501261

[34] Gravina FS, Parkington HC, Kerr KP, de Oliveira RB, Jobling P, Coleman HA, Sandow SL, Davies MM, Imtiaz MS, van Helden DF. Role of mitochondria in contraction and pacemaking in the mouse uterus. Br J Pharmacol. 2010;161:6:1375–90. 10.1111/j.1476-5381.2010.00949.x. 20942856 10.1111/j.1476-5381.2010.00949.x PMC3000661

[35] Webb NE, Montefiori DC, Lee B. Dose–response curve slope helps predict therapeutic potency and breadth of HIV broadly neutralizing antibodies. Nat Commun. 2015;6:18443. 10.1038/ncomms9443. 10.1038/ncomms9443 PMC4588098 26416571

[36] Sampah MES, Shen L, Jilek BL, Siliciano RF. Dose–response curve slope is a missing dimension in the analysis of HIV-1 drug resistance. Proceedings of the National Academy of Sciences. 2011;108:18:7613–7618. 10.1073/pnas.1018360108 10.1073/pnas.1018360108 PMC3088572 21502494

