# Supplementary figures and images for: Assessing Tocolytic Potency: Variability and Accuracy of AUC Versus Amplitude-Based Assessment of Pregnant Human Myometrial Contractions Ex Vivo
Source: Reprod Sci. 2025 Apr 24;32(6):2027–49. doi: 10.1007/s43032-025-01864-0 (PMC12187858; doi:10.1007/s43032-025-01864-0)

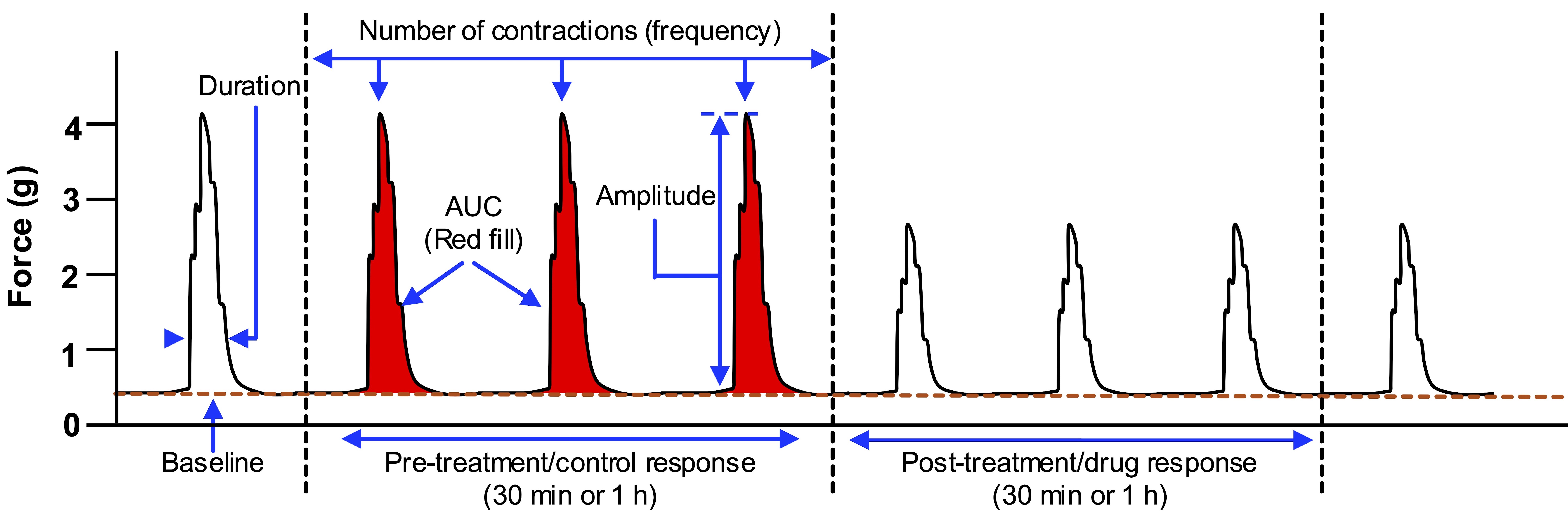

Supplement: Supplementary file 2 — Supplementary Material 2 [file 43032_2025_1864_MOESM2_ESM.jpg]

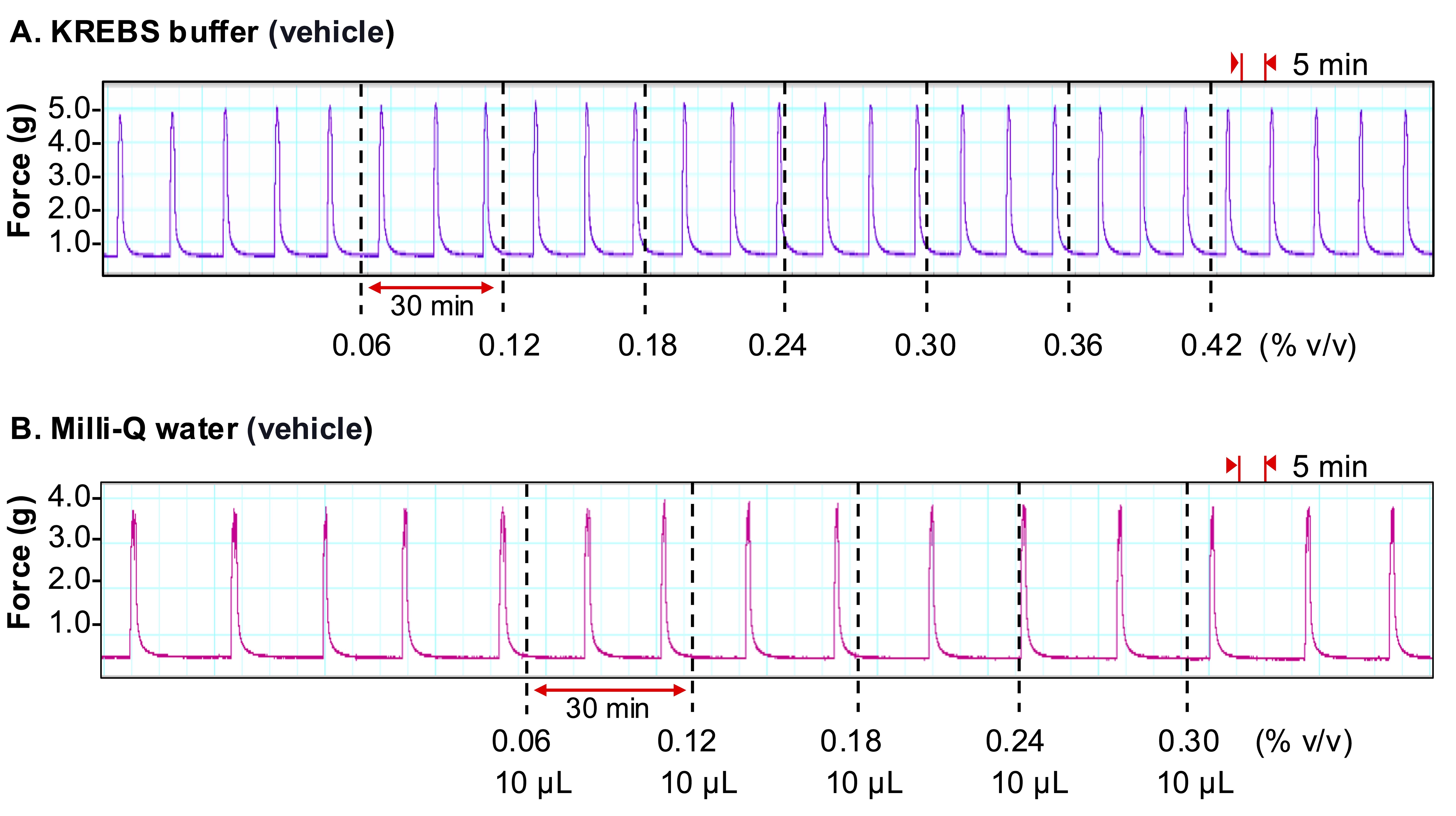

Supplement: Supplementary file 3 — Supplementary Material 3 [file 43032_2025_1864_MOESM3_ESM.jpg]
